# Heterozygous p.Y955C mutation in DNA polymerase γ leads to alterations in bioenergetics, complex I subunit expression, and mtDNA replication

**DOI:** 10.1016/j.jbc.2022.102196

**Published:** 2022-06-24

**Authors:** Md. Mostafijur Rahman, Carolyn K.J. Young, Steffi Goffart, Jaakko L.O. Pohjoismäki, Matthew J. Young

**Affiliations:** 1 Department of Biochemistry and Molecular Biology, Southern Illinois University School of Medicine, Carbondale, Illinois, USA; 2 Department of Environmental and Biological Sciences, University of Eastern Finland, Joensuu, Finland

**Keywords:** mitochondrial DNA (mtDNA) maintenance, *POLG* c.2864A>G/p.Y955C, autosomal dominant progressive external ophthalmoplegia (adPEO), SJCRH30, cell line model of mitochondrial disease, mitochondrial toxicity, 2′-3′-dideoxycytidine (ddC, zalcitabine), adPEO, autosomal dominant progressive external ophthalmoplegia, BCA, bicinchoninic acid ddC, 2′-3′-dideoxycytidine, ddCMP, 2′-3′-dideoxycytidine monophosphate, DIG, digoxigenin, DMU, 1,3-dimethylurea, β, DNA polymerase beta, θ, DNA polymerase theta, ζ, DNA polymerase zeta, dNTP, deoxyribonucleotide triphosphate, DPBS, Dulbecco's phosphate-buffered saline, ds-mtDNA, double-stranded mitochondrial DNA, ECAR, extracellular acidification rate, exo+, exonuclease proficient, exo−, exonuclease deficient, FCCP, carbonyl cyanide p-trifluoromethoxyphenylhydrazone, FBS, fetal bovine serum, gRNA, guide RNA, H-strand, heavy strand, HDR, homology-directed repair, HMW, high molecular weight, IVT, *in vitro* transcription, L-strand, light strand, LMW, low molecular weight, MMW, mid-range molecular weight, mtDNA, mitochondrial DNA, MW, molecular weight, ND4, NADH dehydrogenase subunit 4, nDNA, nuclear DNA, NGS, next-generation sequencing, OCR, oxygen consumption rate, OH, heavy-strand origin of replication, OL, light-strand origin of replication, OXPHOS, oxidative phosphorylation, PMF, proton motive force, Polγ, DNA polymerase gamma, RC, relaxed circular, RI, replication intermediate, SMD, secondary mitochondrial dysfunction, ss-mtDNA, single-stranded mitochondrial DNA, WCE, whole-cell extracted, 1D-AGE, one-dimensional agarose gel electrophoresis, 2DNAGE, two-dimensional neutral agarose gel electrophoresis, 2D-AGE, two-dimensional agarose gel electrophoresis, 2-DG, 2-deoxyglucose

## Abstract

In human cells, ATP is generated using oxidative phosphorylation machinery, which is inoperable without proteins encoded by mitochondrial DNA (mtDNA). The DNA polymerase gamma (Polγ) repairs and replicates the multicopy mtDNA genome in concert with additional factors. The Polγ catalytic subunit is encoded by the *POLG* gene, and mutations in this gene cause mtDNA genome instability and disease. Barriers to studying the molecular effects of disease mutations include scarcity of patient samples and a lack of available mutant models; therefore, we developed a human SJCRH30 myoblast cell line model with the most common autosomal dominant *POLG* mutation, c.2864A>G/p.Y955C, as individuals with this mutation can present with progressive skeletal muscle weakness. Using on-target sequencing, we detected a 50% conversion frequency of the mutation, confirming heterozygous Y955C substitution. We found mutated cells grew slowly in a glucose-containing medium and had reduced mitochondrial bioenergetics compared with the parental cell line. Furthermore, growing Y955C cells in a galactose-containing medium to obligate mitochondrial function enhanced these bioenergetic deficits. Also, we show complex I NDUFB8 and ND3 protein levels were decreased in the mutant cell line, and the maintenance of mtDNA was severely impaired (*i.e.*, lower copy number, fewer nucleoids, and an accumulation of Y955C-specific replication intermediates). Finally, we show the mutant cells have increased sensitivity to the mitochondrial toxicant 2′-3′-dideoxycytidine. We expect this *POLG* Y955C cell line to be a robust system to identify new mitochondrial toxicants and therapeutics to treat mitochondrial dysfunction.

Cells synthesize most of their ATP using the mitochondrial oxidative phosphorylation (OXPHOS) machinery, and this machinery requires 13 mitochondrial DNA (mtDNA)-encoded proteins to function. Thus, mtDNA maintenance is essential to meeting the basic energy demands within our cells. The human mtDNA genome is a covalently closed circular double-stranded 16,569-bp molecule that harbors the above-mentioned 13 OXPHOS genes in addition to 2 genes encoding rRNAs and 22 genes coding for tRNAs. The 24 RNA genes are required to translate the 13 mtDNA-encoded polypeptides. Disease mutations are associated with all 37 mtDNA genes, and 1 in 200 healthy individuals harbors a pathogenic mtDNA mutation that could cause disease in a child born to a female carrier (1, 2), underscoring the importance of maintaining the mtDNA genome.

The multicopy mtDNA genome is replicated and repaired throughout the cell cycle by the mtDNA polymerase gamma (Polγ) in concert with additional replisome factors, for example, Twinkle mtDNA helicase, topoisomerases, mitochondrial single-stranded DNA-binding protein, and others (3). According to the strand displacement model of mtDNA replication, replisomes containing Polγ synthesize both the nascent heavy (H) and light (L) strands continuously without the formation of Okazaki-fragment-like replication products (4). The two mtDNA strands are named H and L based on the ability to separate them on denaturing cesium chloride gradients. The H-strand is richer in G + T content, making it heavier on density centrifugation (5, 6). The Polγ holoenzyme is composed of three subunits encoded by two nuclear DNA (nDNA) genes: (1) *POLG* codes for the 140-kDa catalytic subunit, p140 (or Polγα), and (2) *POLG2* encodes the ∼110-kDa homodimeric accessory subunit, p55 (or Polγβ). Mutations in *POLG* and *POLG2* are associated with primary mitochondrial disorders that cause mtDNA instability, such as mtDNA depletion and deletions. *POLG* mutations are the most common cause of inherited mitochondrial disease, and the number of individuals harboring a *POLG* mutation is estimated to be ∼2% of the population (7).

The most common autosomal dominant *POLG* mutation is the c.2864A>G substitution, which encodes a substitution of the p140 tyrosine (Tyr/Y) 955 amino acid residue for cysteine (Cys/C), p140 Y955C (8). In a groundbreaking 2001 paper, the heterozygous *POLG* c.2864A>G/p.Y955C mutation (hereafter *POLG* Y955C) cosegregated with autosomal dominant progressive external ophthalmoplegia (adPEO) in a family, establishing that a *POLG* mutation causes the disorder (9). Manifestation of adPEO is characterized by adult-onset progressive weakness of the extraocular eye muscles resulting in strabismus and ptosis (7, 8). In addition, muscle weakness can extend to the limb-girdle skeletal muscles (manifesting as generalized myopathy or proximal myopathy). Other symptoms include parkinsonism, ataxia, sensorineural hearing loss, depression, cataracts, and premature ovarian failure (8). Unrelated patients with *POLG* Y955C mutations harbor mtDNA deletions in skeletal muscle samples and ∼20% to ∼60% reductions in mtDNA content relative to control subjects (9, 10, 11, 12). In a study where p140 Y955C was overexpressed in 293 Flp-In TRex cells, mtDNA levels were reduced by >60% (13).

Several biochemical and biological models have been developed to understand the molecular mechanisms contributing to mitochondrial dysfunction associated with adPEO. However, due to the scarcity of patient samples, the molecular and physiological details regarding the effects of adPEO mutations on human cell mtDNA homeostasis, bioenergetics, and OXPHOS machinery remain poorly understood. Based on previous biochemical, crystal structure, and molecular model work, the Y955C p140 substitution has been demonstrated to localize at the Polγ DNA polymerase active site and cause significantly reduced dNTP incorporation. Still, the Y955C variant enzyme maintains wild-type (WT)-like DNA-binding (14, 15, 16, 17, 18). Additionally, the 3′-5′ exonuclease (exo+), or proofreading proficient, Y955C Polγ holoenzyme is 2-fold less accurate than WT Polγ despite having a functional exo+ domain (14, 19). Intriguingly, in another study, when provided a high concentration of dATP (200 μM) and 1 μM other dNTPs, Y955C Polγ DNA synthesis activity could be restored. The authors elegantly showed that when WT and Y955C Polγ holoenzymes are mixed using a constant concentration of dNTPs (1 μM), Y955C Polγ is dominant-negative, and *via* its exo+ activity, chews back nascently synthesized DNA and likely stalls on the template in an idling mode, which blocks the activity of WT Polγ (13). In agreement with an autosomal dominant mode of inheritance, an *in vitro* rolling circle replication assay demonstrated that the Y955C Polγ holoenzyme has a dominant-negative effect on DNA synthesis in the presence of the WT Polγ holoenzyme at dNTP concentrations of 1 and 10 μM (10).

In yeast models harboring the orthologous *POLG* Y955C mutation (p.Y757C), haploid yeast suffered a complete loss of the mtDNA genome and subsequently the ability to respire and maintain cellular viability. Diploid yeast harboring the p.Y757C heterozygous variant had severely reduced mtDNA copy number (about half of WT levels) and reduced respiratory competence with a significant increase in mtDNA damage (20). Another study used a heteroallelic yeast strain harboring both the WT *POLG* (*MIP1*) and p.Y757C mutant alleles to show that increasing dNTP pools (by overexpression of the *RNR1* ribonucleotide reductase gene) suppresses mtDNA damage and depletion (21). In *Drosophila melanogaster* lines, homozygous for the orthologous *POLG* Y955C mutation (p.Y873C), larval lethality at the third instar stage was seen, but heterozygous p.Y873C flies did not show phenotypic abnormalities or mtDNA deletions. Although heterozygous p.Y873C flies had normal lifespans after 15 generations of intercrossing, the flies were developmentally delayed and presented with mtDNA depletion (∼20% less mtDNA compared with WT flies at the F1 generation) and ∼40% reduced from WT at the F15 generation (10). Also, *POLG* Y955C heart-specific transgenic overexpression in a mouse model caused cardiomyopathy with decreased mtDNA (about half of WT levels) and mitochondrial ultrastructural defects (22).

Barriers to studying mitochondrial dysfunction include scarcity of patient-derived fibroblasts, scarcity of patient tissue samples, and a lack of readily available human cell line models that harbor disease-causing mutations. Many disorders have a ‘mitochondrial phenotype’ but lack a mtDNA or nDNA mutation suggesting many unidentified or hard to detect exogenous factors play a role in secondary mitochondrial dysfunction (SMD). Also, SMD is associated with many other diseases, including fatty acid oxidation disorders, limb-girdle muscular dystrophy, myopathy, drug-induced peripheral neuropathies, spinal muscular atrophy, cancer, and many others (23). We suspect that a human cell line model harboring a mitochondrial disease mutation will have enhanced sensitivity to mitochondrial stressors, thereby allowing the detection of agents that otherwise may be undetectable using standard WT cell lines.

Here we engineered a knock-in of the *POLG* Y955C heterozygous substitution into the nuclear genome of the human SJCRH30 cell line using CRISPR-Cas9. The SJCRH30 cell line is derived from the tumor of a 17-year-old male with rhabdomyosarcoma. SJCRH30 cells harbor attenuated sarcomere structures resembling those found in primitive rhabdomyoblasts (24). SJCRH30 has been used to evaluate the cytotoxicity of chemotherapeutic drugs such as cisplatin, doxorubicin, and topotecan (25, 26). Also, SJCRH30 has been used as a model of human myoblasts to study the regulation of mitochondrial biogenesis and cellular oxygen consumption rates (27, 28). The effects of the *POLG* Y955C mutation on cell growth, mtDNA maintenance, OXPHOS machinery subunits, cellular bioenergetics, and sensitivity to the known mitochondrial toxicant 2′-3′-dideoxycytidine (ddC) were determined.

## Results

### Generation of the SJCRH30 *POLG* Y955C cell line

**Figure 1:**
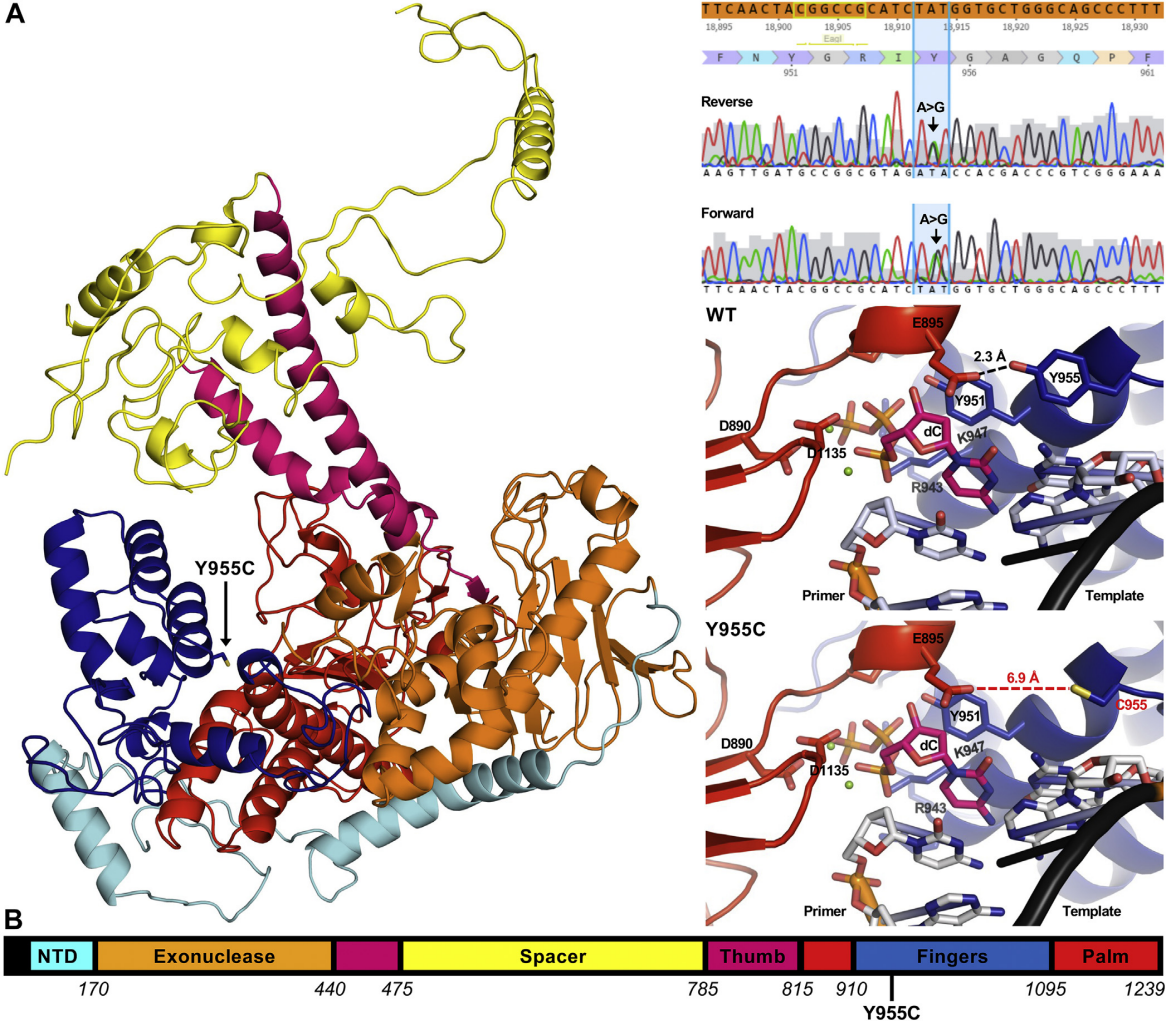
**Substitution of the p140 tyrosine 955 amino acid residue for cysteine (Y955C) likely disrupts a key intramolecular interaction between the palm and fingers subdomains.***A*, *left*, model of the p140 Y955C variant. The p140 DNA polymerase domain folds to resemble a “*right hand*” composed of the palm (*red*), fingers (*blue*), and thumb (*magenta*) subdomains. Other regions include the aminoterminal domain (NTD; *cyan*), the spacer domain (*yellow*), and the exonuclease domain (*orange*). Note that the Y955C substitution localizes to the finger’s subdomain. The model was generated using the PDB ID 4ZTZ structure as a template in Missense3D (100). *Top right*, Sanger sequencing traces of both strands of a *POLG* exon 18 PCR product confirming the heterozygous *POLG* c.2864A>G/p.Y955C mutation. *Middle right* and *bottom right*, wildtype (WT) and p140 Y955C active sites, respectively. D890 and D1135 are key catalytic residues that interact with the dCTP (dC, *colored pink*)-magnesium (*green spheres*) complex. R943, K947, and Y951 of the O-helix (residues 943–955) coordinate dC at the active site. Y955 is vital for effective dNTP binding and the pronounced template bending in the single-/double-strand junction. Y955 is predicted to make an intramolecular hydrogen bond with E895, and this interaction is lost in Y955C. Nitrogens are *colored blue*, oxygens *light red*, *sulfur yellow*, and phosphates *orange*. *B*, linear representation of the p140 catalytic subunit amino acid residues. Colors are as described in *A*.

Before gene editing, studies of genetic disease mutations relied on primary cells, and assessment of patient pathophysiology can require post mortem or *in vivo* measurement such as MRI. Therefore, our goal was to engineer a cell line model harboring the *POLG* Y955C variant to have an unrestricted number of cells to understand better the cellular and molecular impacts of this mitochondrial disease mutation. The SJCRH30 cell line was transfected with an *in vitro* transcription (IVT) guide RNA (gRNA), the GeneArt TrueCut Cas9 V2 nuclease, and a donor single-stranded DNA (ssDNA) oligonucleotide harboring the *POLG* Y955C mutation as described under *Experimental Procedures*. Isolated clones were screened by cell lysis plus PCR amplification of a specific flanking *POLG* Y955C region, and the PCR products were sequenced by Sanger sequencing. A candidate clone from the Sanger sequencing analysis was subjected to on-target next-generation sequencing (NGS) analysis. NGS confirmed a single clone with 50% homology-directed repair (HDR), indicating that the clone harbored a heterozygous *POLG* Y955C mutation. Following the expansion of the clone, the mutation was reverified by on-target NGS and Sanger sequencing, Figure 1*A*. Maintenance of the Y955C heterozygous locus was checked and verified again at the end of the study using Sanger sequencing.

Flow cytometry of propidium iodide–stained cells helps measure cellular DNA and segregates cells into their cell cycle phases (29, 30). Therefore, we performed cell cycle flow cytometry analysis on *POLG* Y955C cells and determined that the cell cycle stages and ploidy were like that of the SJCRH30 parental cell line, Fig. S1. Our results support that a *POLG* Y955C heterozygous mutation was sufficiently inserted into the SJCRH30 nuclear genome.

### The p140 p.Y955C variant is predicted to disrupt a key intramolecular interaction

Based on the previously published crystal structure of Polγ bound to a primer-template, Y955 is located at the end of an alpha helix, which also contains the crucial Y951 residue that stacks with the incoming nucleotide, Figure 1*A*. Y955, along with R943 and K947, assist with facilitating the effective binding of an incoming nucleotide into the p140 active site. Furthermore, the p140 Y955 residue is needed for the bending of DNA located in a single double strand junction. This bending is mediated by Y955 and a short connecting loop that contains amino acid residues A957 and G958 (15). The Y955 and E895 side chains are in a close 2.3 Å proximity to each other. We predict the Y955 side chain hydroxyl group forms a critical intramolecular hydrogen bond with the E895 side chain carbonyl group. Y955 is in the finger’s subdomain of p140 (Fig. 1*B*), while E895 is located in the palm subdomain. Y955C is predicted to disrupt the close 2.3 Å interaction between residues at 955 and 895, resulting in the distance between C955 and E895 increasing to 6.9 Å in the model. We expect that disruption of this hydrogen bond is detrimental to Polγ and results in a destabilization of the active site conformation, which prevents the efficient incorporation of nucleotides into nascent mtDNA.

### The SJCRH30 *POLG* Y955C cell line has a slow growth phenotype

**Figure 2:**
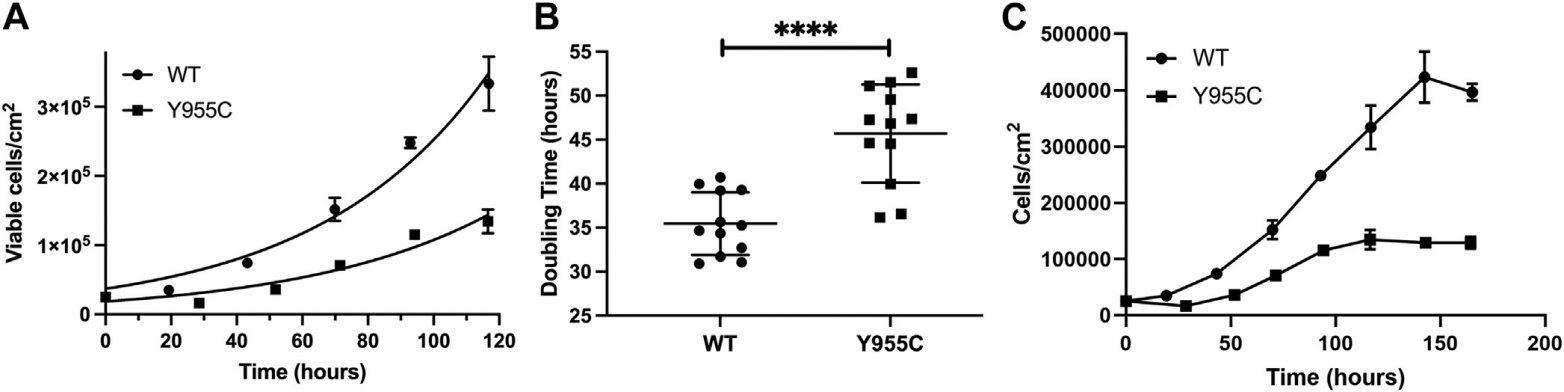
**SJCRH30 *POLG* Y955C cells grow more slowly than wildtype cells under standard tissue culture conditions.***A*, *POLG* Wildtype (WT) and Y955C growth curves. Exponential growth was observed for both cell types. After 5 days of growth, the number of viable WT cells was ∼2.5-fold higher than the mutant. Mean viable cells/cm^2^ values (n = 4) and standard deviation (SD) errors for a representative experiment (P17 for WT and P8 for Y955C) are shown in the graph. *B*, doubling time of *POLG* WT and Y955C cells. The mean doubling times (DT) based on three independent experiments utilizing WT at passages 13, 15, and 17, and Y955C at 8, 10, and 11 are reported in hours with error as SDs. The mean doubling time of WT cells is significantly less than Y955C cells, ∗∗∗∗*p* < 0.0001, as judged by a Student’s *t* test. DT values were calculated using the least-squares fit of the exponential growth equation in Graph Pad Prism. *C*, *POLG* WT and mutant cell densities (cells/cm^2^) up to 7 days of growth. Data are from the same representative experiment shown in *A*.

The doubling times of the SJCRH30 *POLG* WT and Y955C cell lines were calculated from the cell counts monitored over 5 days to determine whether the *POLG* Y955C mutation affects cell growth (Fig. 2*A*). The mean doubling times of the WT and Y955C mutant were 35.5 ± 3.6 and 45.7 ± 5.6 h, respectively, and the decreased growth rate for Y955C is significantly different, Figure 2*B*. By day 7, the average Y955C viable cell densities remaining on the tissue culture dishes were ∼3.1-fold less than WT (Fig. 2*C*). Also, both cell types maintained >95% cellular viability over the 7-day timeline as judged by the trypan blue exclusion method.

### The SJCRH30 *POLG* Y955C cell line has compromised bioenergetic parameters

**Figure 3:**
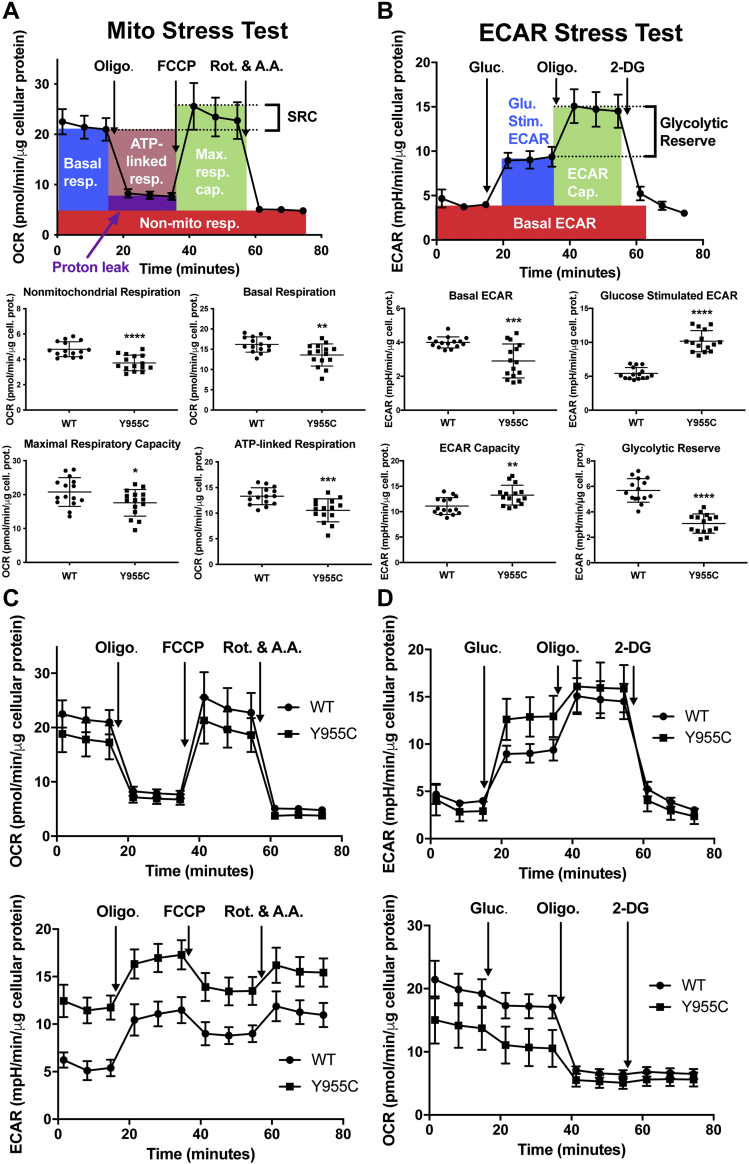
**SJCRH30 *POLG* Y955C cells have altered bioenergetics.***A*, results from Mito Stress tests comparing SJCRH30 *POLG* wildtype (WT) and Y955C mutant cells side-by-side on XFp cell culture miniplates. *Top*, Description of mitochondrial bioenergetic parameters: basal respiration (Basal resp.), ATP-linked respiration (ATP-linked resp.), maximal respiratory capacity (Max. resp. cap.), spare respiratory capacity (SRC), proton leak, and nonmitochondrial respiration (Non-mito resp.). Metabolic stressors are injected sequentially from Ports A (oligomycin), B (FCCP), and C (antimycin A + rotenone). *Bottom*, Mito Stress test bioenergetic parameters. *B*, results from ECAR Stress tests comparing WT and Y955C mutant cells side-by-side on XFp cell culture miniplates. *Top*, Description of ECAR bioenergetic parameters: basal ECAR, glucose-stimulated ECAR (Glu. Stim.), ECAR capacity (ECAR Cap.), and apparent glycolytic reserve (Glycolytic Reserve). First, glucose was injected from port A, followed by oligomycin (port B), and 2-deoxyglucose (port C). *Bottom*, ECAR Stress Test bioenergetic parameters. Complete OCR and ECAR profiles for WT and Y955C *C*, Mito Stress and *D*, ECAR Stress Tests. Data in the graphs and scatter plots are presented as mean ± SD, n = 15 (in triplicate or sextuplicate from three independent experiments using different passages). For several data points in the top panels in A and B, errors are not shown as the error bars are shorter than the height of the symbol. Oligo., oligomycin; Rot., rotenone; A.A., antimycin A; Gluc., glucose; 2-DG, 2-deoxyglucose. ∗∗∗∗*p* ≤ 0.0001, ∗∗∗*p* ≤ 0.001, ∗∗*p* ≤ 0.01, and ∗*p* ≤ 0.05. Statistical significance between two parametric groups was determined using a Student’s or a Welch's *t* test, while significance between two nonparametric groups was determined using a Mann–Whitney U test.

To determine the downstream effects of the *POLG* Y955C mutation on SJCRH30 mitochondrial bioenergetic profiles *POLG* WT and Y955C cells were exposed to known pharmacological stressors of the OXPHOS machinery. Mitochondrial OXPHOS is the oxygen-dependent process of coupling substrate oxidation to produce the energy-rich molecule adenosine triphosphate (ATP). The 37 mtDNA genes are required for OXPHOS, and mitochondrial translation of the 13 mtDNA-encoded polypeptides generates essential OXPHOS machinery subunits. During OXPHOS, molecular oxygen (O_2_) is reduced to water (H_2_O). Using the Seahorse XFp extracellular flux analyzer, O_2_ biosensors measure the real-time rate at which cells convert O_2_ to H_2_O, the O_2_ consumption rate (OCR). The second set of XFp biosensors measure the extracellular acidification rate (ECAR) resulting from the cytoplasmic breakdown of glucose-derived pyruvate to lactate and the respiratory evolution of carbon dioxide (CO_2_). Glycolysis is the major cytosolic O_2_-independent metabolic pathway that converts one glucose molecule into two molecules of pyruvate, ATP, and NADH. When pyruvate is shunted through the mitochondrion to the pyruvate dehydrogenase complex, and subsequently through the tricarboxylic acid cycle, CO_2_ is generated. In solution, a molecule of CO_2_ can combine with a molecule of H_2_O forming carbonic acid that dissociates at physiological pH into the bicarbonate anion and a proton that contributes to medium acidification. In a Mito Stress test, oligomycin, carbonyl cyanide p-trifluoromethoxyphenylhydrazone (FCCP), and rotenone + antimycin A are sequentially injected to inhibit ATP synthase (OXPHOS complex V), dissipate the mitochondrial proton motive force, and inhibit OXPHOS complex I + III, respectively. Six Mito Stress test bioenergetic parameters are determined using these four stressors. Rotenone plus antimycin A is added last during the experiment to terminate electron flow through the electron transport chain and enable calculation of the OCR from nonmitochondrial oxidases (*nonmitochondrial respiration*). This nonmitochondrial OCR is subtracted to accurately determine mitochondrial contributions to *basal respiration* (baseline OCR), *proton leak–linked respiration* (the remaining respiration in the presence of oligomycin), and *maximal respiratory capacity* (a measure of the ability of a protonophore, FCCP, to uncouple proton movement and ATP synthesis, and restore flux through complexes I–IV). *ATP-linked respiration* measures the OCR coupled to ATP production, and spare respiratory capacity is the difference in OCRs between maximal respiratory capacity and basal respiration. Spare respiratory capacity is defined as the extramitochondrial capacity available to produce ATP during increased work or stress conditions (31, 32). Compared with WT, Y955C has significantly decreased mitochondrial function as indicated by decreases in basal respiration (16% reduced), ATP-linked respiration (21% reduced), and maximal respiratory capacity (15% reduced), Figure 3*A*. Also, Y955C nonmitochondrial respiration was significantly decreased by 23%.

Interestingly, enhanced *POLG* Y955C ECAR relative to WT was observed during all stages of the Mito Stress test (see the bottom panel of Fig. 3*C*). ECARs increased in both cell types following oligomycin injection, suggesting the glycolytic pathway compensates for the block at OXPHOS complex V. Also, at later time points, the slight but significant increase in ECAR values in both cell types following injection of rotenone plus antimycin A suggests the enhanced ECAR results from the glycolytic pathway and not from the tricarboxylic acid cycle activity (bicarbonate-associated acid production), *i.e.*, compare the differences between the 12th and 9th time points in both cell types at ∼75 and ∼55 min, *p* < 0.0001 for WT (11.0 ± 1.3 and 9.0 ± 0.9 mpH/minute/μg cellular protein, respectively) and *p* < 0.0014 for Y955C (15.4 ± 1.5 and 13.5 ± 1.5 mpH/minute/μg cellular protein, respectively) (31, 32). Therefore, the glycolytic pathway is likely active in both cell types, and we hypothesized that glycolysis is upregulated in *POLG* Y955C to compensate for compromised OXPHOS.

To further investigate the difference in ECARs observed between the two cell types, we conducted ECAR Stress tests. ECAR Stress tests sequentially inject glucose, oligomycin, and 2-deoxyglucose (2-DG) into a glucose-free medium, bathing WT or Y955C cells. Following glucose injection, oligomycin inhibits the OXPHOS machinery and allows estimation of ECAR parameters. As a competitive hexokinase inhibitor, 2-DG inhibits the cell’s ability to utilize free glucose to generate pyruvate *via* glycolysis, and ECAR drastically decreases (33). *Basal ECAR* is the last of three ECARs measured immediately before sugar injection (∼15 min into the ECAR Stress test). *Glucose-stimulated ECAR* is the maximal rate following glucose injection (but before oligomycin injection) minus basal ECAR. Glucose-stimulated ECAR represents the total extracellular acidification from cellular pathways metabolizing free glucose, including the production of cytoplasmic lactate and mitochondrial protons produced *via* CO_2_ hydration and dissociation. However, as we observed an increase in ECAR values in both SJCRH30 cell types following oligomycin and rotenone + antimycin A injections in Mito Stress tests, *glucose-stimulated ECAR can serve as a proxy for glycolysis ECAR*. *ECAR capacity* (glycolytic capacity) is a measurement of extracellular acidification, including glucose-stimulated ECAR and oligomycin-stimulated ECAR production. The *apparent glycolytic reserve* parameter is obtained by subtracting glucose-stimulated ECAR from ECAR capacity. The apparent glycolytic reserve is the estimated amount of unused glycolytic capability of the cell that could be utilized if cellular ATP demand was increased. In the absence of glucose, *POLG* Y955C cell basal ECAR was ∼30% lower than WT. Following glucose injection, Y955C glucose-stimulated (glycolytic) ECAR increased 1.9-fold compared with WT, suggesting that mutant cells rely more heavily on the glycolytic pathway to generate ATP. In addition, Y955C ECAR capacity was 1.2-fold higher than WT when OXPHOS is shut down by oligomycin. Still the apparent glycolytic reserve capacity was only about half of WT levels indicating a reduced glycolytic capability with increased energy demand, Figure 3, *B* and *D*.

**Figure 4:**
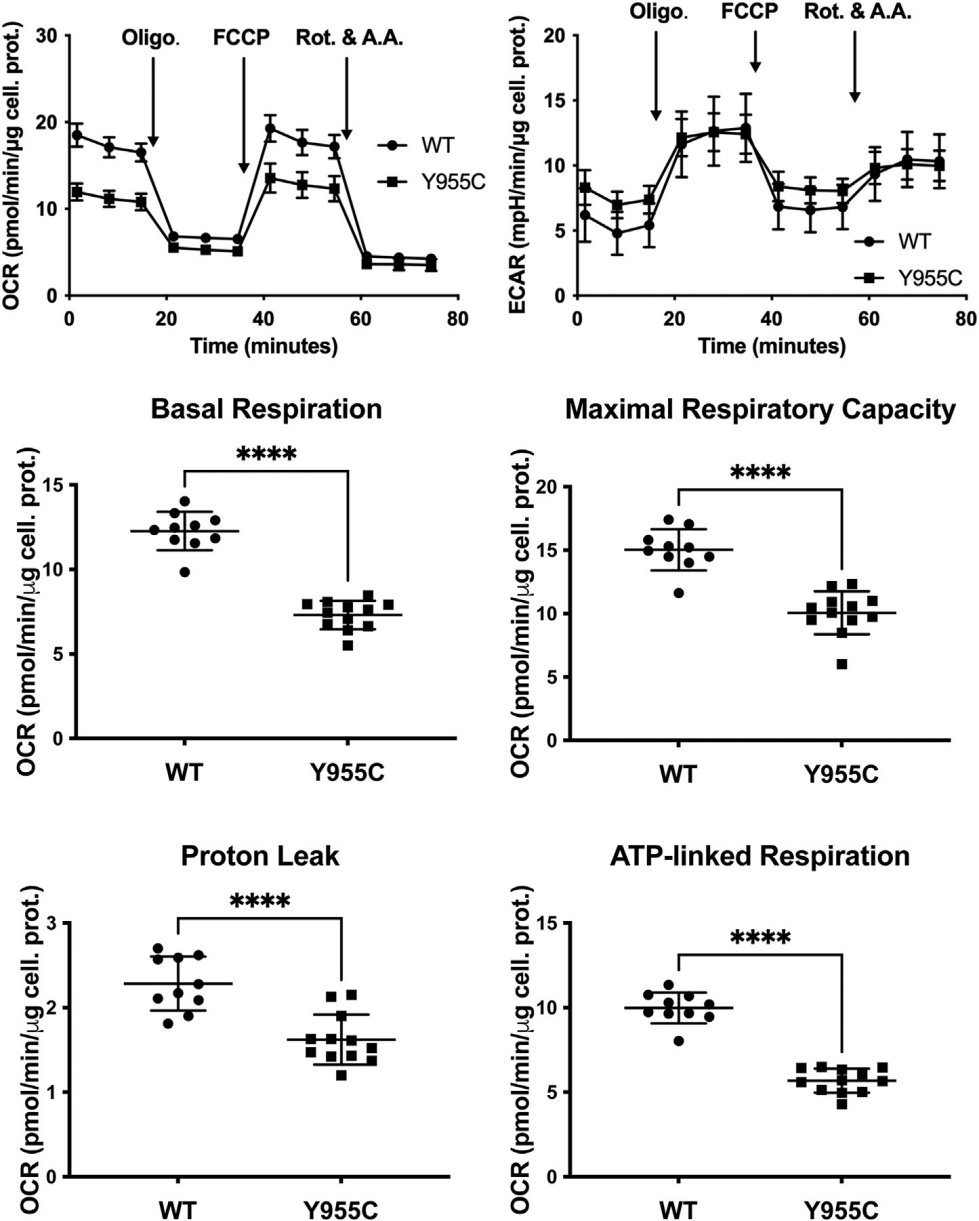
**SJCRH30 *POLG* Y955C mitochondrial bioenergetic deficiencies are enhanced when galactose is substituted for glucose in the growth medium.** Results from Mito Stress tests comparing *POLG* wildtype (WT) and Y955C mutant cells are shown. Data in the graphs and scatter plots are presented as mean ± SD, n ≥ 10 (from three independent experiments using different passages). For several data points in the *top left panel*, errors are not shown as the error bars are shorter than the *height* of the symbol. Abbreviations are as described in Figure 3. ∗∗∗∗*p* ≤ 0.0001. Statistical significance was determined using Student’s *t* tests.

As skeletal muscle cells heavily rely on anaerobic glycolysis to generate ATP during muscle contraction (34), we performed Mito Stress tests following cell growth in a galactose-based medium to force the cells to rely on mitochondrial OXPHOS (35) and to more clearly understand the effect of the *POLG* Y955C mutation on mitochondrial bioenergetics. Indeed, when compared with cells grown in glucose (Fig. 3), galactose-grown *POLG* Y955C cells have >2-fold reductions in basal respiration (41% reduced compared with WT), maximal respiratory capacity (33% reduced), and ATP-linked respiration (43% reduced), Figure 4. The nonmitochondrial respiration changes were similar in the two experiments (19% reduced in galactose-grown Y955C cells *versus* 23% reduced in glucose-grown cells). Still, a 30% decrease in Y955C proton leak is apparent under galactose conditions. Furthermore, the significantly higher Y955C Mito Stress test ECARs observed in the glucose-grown cells (Fig. 3) are not seen in the galactose-grown cells (Fig. 4), supporting the concept that growth in galactose obligates OXPHOS function. Based on these results, experiments performed in glucose-grown SJCRH30 cells can omit the effects of the *POLG* Y955C mutation on proton leak and weaken the fold-change results of mitochondrial bioenergetics.

### OXPHOS complex I subunit protein expression levels are decreased in *POLG* Y955C cells

**Figure 5:**
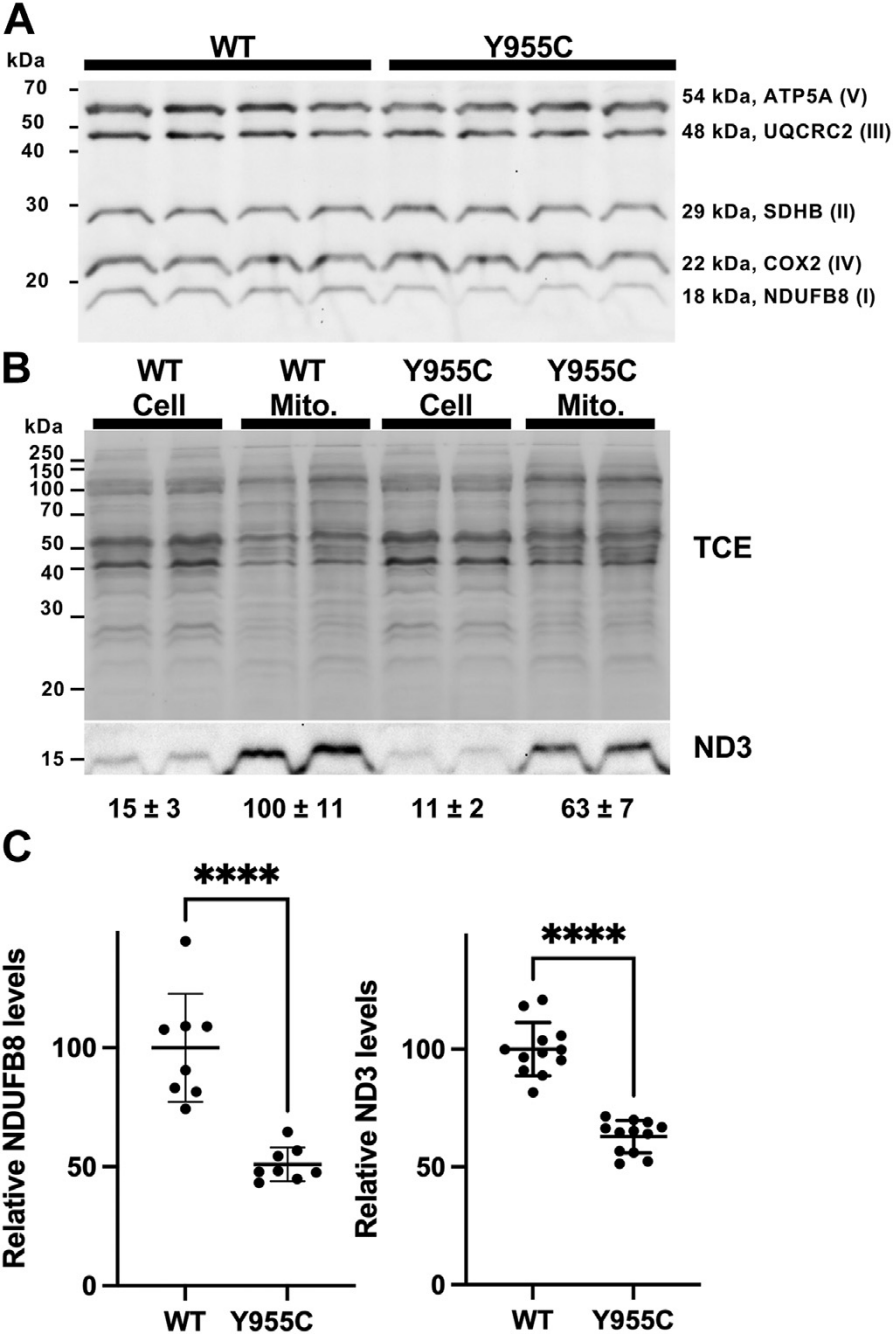
**Mitochondrial NADH dehydrogenase complex I NDUFB8, and ND3 levels are decreased in *POLG* Y955C.***A*, Y955C NDUFB8 expression is decreased compared with WT. *POLG* wildtype (WT) and Y955C mitochondrial extracts were run on SDS-PAGE, followed by Western blot analysis. An antibody cocktail containing monoclonal antibodies specific for a subunit of each OXPHOS complex (I, II, III, IV, and V) was used. Four of the subunits are encoded by the nuclear genome (ATP5A, UQCRC2, SDHB, and NDUFB8), and one is encoded by the mtDNA genome (COX2). A representative blot is shown. *B*, in comparison with WT, Y955C ND3 expression is decreased. *POLG* WT and Y955C mitochondrial (Mito.) and whole-cell (Cell) extracts were subjected to Western blot analysis as described in *A*, but a mtDNA encoded ND3-specific polyclonal primary antibody was used. The trichloroethanol (TCE)–stained whole-cell or whole-mitochondrial protein in each lane of the blot was used to normalize the data for the chemiluminescent bands, and a representative blot is shown. *C*, relative expression levels of NDUFB8 and ND3 in mitochondrial protein extracts. The chemiluminescent band areas in each lane were normalized to their respective total protein signal on the blot. The WT values were set to 100%, and the data are mean ± SDs. For the NDUFB8 subunit, the experiment was repeated twice on different days (n = 8 lanes from two blots), and for the ND3 subunit, the experiment was repeated in triplicate on different days (n = 12 from six blots; two blots per experiment). ∗∗∗∗*p* ≤ 0.0001.

As mentioned earlier, *POLG* Y955C is associated with mtDNA depletion and deletions in patient skeletal muscle samples (9, 10, 11, 12). Therefore, we hypothesized that, in a *POLG* Y955C cell, the mitochondrial OXPHOS machinery will be disrupted or depleted as 13 essential OXPHOS subunits are encoded by the mtDNA. Indeed, using samples from postmortem patients with *POLG*-related disorders, substantia nigra neurons have been demonstrated to have decreased expression of OXPHOS complex I or IV subunits or both, *e.g.*, 1. *POLG* p.Gly848Ser and p.Ser1104Cys, 2. p.Trp748Ser and p.Arg1096Cys, 3. p.Ala467Thr and p.Trp748Ser, and 4. p.Thr251Ile and p.Ala467Thr (36). Interestingly, a nuclear-encoded complex I subunit was used to measure the decreased expression (NDUFB8) of the complex, suggesting that disruption of expression of one or more of the mtDNA-encoded complex I subunits in *POLG*-related disease (ND1, ND2, ND3, ND4L, ND4, ND5, ND6) destabilize the complex leading to destabilization or proteolysis and degradation of the respirasome and supercomplexes. The respirasome and supercomplexes are higher-order supramolecular structures composed of OXPHOS machinery enzyme complexes (37, 38). Therefore, to determine whether the OXPHOS machinery is disrupted in *POLG* Y955C cells, we isolated mutant and *POLG* WT mitochondria and then analyzed a subunit from each OXPHOS complex *via* Western blotting. Using this approach, an ∼2-fold decrease in expression of the NADH dehydrogenase complex I NDUFB8 subunit was detected in the Y955C mutant, but the other complex subunits remained at WT levels, Figure 5. Although the expression level of the Y955C mtDNA-encoded cytochrome c oxidase complex IV COX2 subunit was not different from WT (Fig. 5*A*), we speculated that disruption of expression of a mtDNA-encoded complex I subunit(s) might lead to destabilization and degradation of the nuclear-encoded NDUFB8 subunit. The protein expression level of the ND3 subunit was investigated to test this idea. Indeed, Y955C mitochondrial ND3 was 1.6-fold decreased compared with WT, Figure 5, *B*, and *C*.

### Restriction enzyme mapping shows *POLG* Y955C mtDNA harbors expected fragment sizes

**Figure 6:**
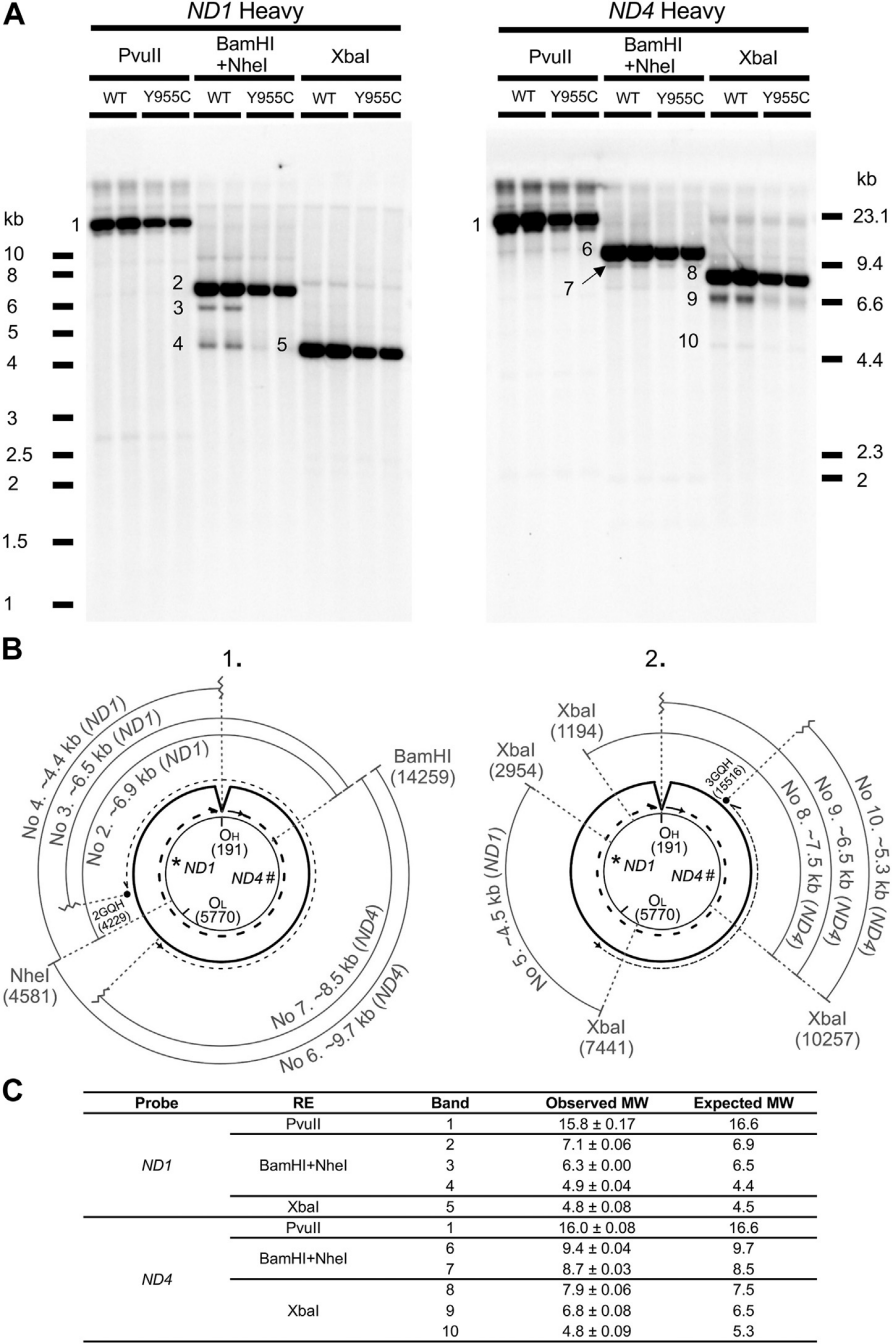
**Restriction endonuclease mapping of SJCRH30 *POLG* WT and Y955C mtDNA.***A*, detection of mtDNA restriction fragments with either an *ND1* heavy (H)-strand (*left*) or *ND4* H-strand (*right*) single-stranded (ss) DIG-labeled probe. *B*, maps of the expected mtDNA restriction fragment lengths when visualized with either the *ND1* or *ND4* probe. Panel 1 shows expected fragment lengths produced with BamHI/NheI double digestion, and panel 2 shows expected lengths produced with XbaI digestion. In both panels, the mtDNA light (L) strand is represented as a thin intact *inner circle* and the H-strand origin of replication (O_H_), the L-strand origin of replication (O_L_), and the *ND1* (∗) and *ND4* (#) probe sequences are also highlighted on this strand. Note that the numbering of base pairs is counterclockwise and is based on mtDNA NC_012920. Nascent continuous leading H-strand synthesis, using the L-strand template and beginning at O_H_ (position 191), is represented as a thick *dashed line* with replication proceeding clockwise around the *circle*. The parental/template H-strand is shown as a displaced intact, *thick circle* that is used as the template strand during continuous nascent lagging L-strand synthesis, which is represented as a thin *dashed line* beginning at O_L_ (position 5770) and proceeding counterclockwise. The location of two predicted G-quadruplexes (G4s) are shown in panels 1 and 2 as *black circles* above the parental H-strand, 2GQH located at positions 4260 to 4229, and 3GQH located at 15545 to 15516. *C*, summary of the expected and estimated molecular weights (MW) of mtDNA bands generated by restriction endonuclease (RE) digestions. PvuII-digested mtDNA fragment lengths greater than 10 kb were estimated using linear regression of the log10 base pair values of Lambda DNA/HindIII Marker 2 fragments loaded onto the same gel *versus* distance traveled in millimeters (R^2^ values were ≥ 0.94). For the remaining restriction digests that generate fragment lengths less than 10 kb, linear regression of the log10 base pair values of exACTGene 1 kb Plus DNA ladder fragments *versus* distance traveled in millimeters was utilized to estimate molecular weights (R^2^ values were ≥ 0.99).

Southern blotting and mtDNA-specific probes to visualize restriction endonuclease digestion products are powerful tools for identifying mtDNA deletions and replication intermediates (39, 40). The two mtDNA strands are named heavy (H) and light (L) based on the ability to separate them on denaturing cesium chloride gradients (5, 6). The origin of H-strand DNA replication (O_H_) is located in the noncoding control region, and the origin of L-strand replication (O_L_) is situated at ∼11,000 base pairs downstream of O_H_ (4). O_H_ and O_L_ divide the circular mtDNA genome into the major arc, which harbors the NADH dehydrogenase subunit 4 gene (*ND4*), and the minor arc, which contains the *ND1* gene. Many mtDNA deletions localize within the mtDNA major arc (41, 42, 43). To determine whether the SJCRH30 *POLG* Y955C cell line harbors mtDNA deletions, WT and Y955C mtDNA restriction maps were analyzed using key restriction enzymes and two single-stranded (ss) oligonucleotide probes, *ND4* and *ND1*. PvuII cuts once in the mtDNA genome and linearizes covalently closed circular mtDNA to a discrete band of ∼16.6 kb, Figure 6*A*. Both BamHI and NheI cut once within the covalently closed circular ds-mtDNA, and a double digest reaction with these enzymes generates ∼9.7- and ∼6.9-kb linear fragments. Also, XbaI was used and cuts 5 times in the mitochondrial genome creating five linear fragments that are ∼1.8, ∼4.5, ∼0.9, ∼2.0, and ∼7.5 kb in size (note, only four XbaI cut sites are shown in the map in Fig. 6*B* to emphasize the bands detected with the two mtDNA-specific probes).

As mtDNA molecules harboring deletions still usually harbor the *ND1* gene (42), an *ND1* minor arc–specific probe should detect molecules harboring deletions when linearized with a restriction endonuclease. The expected WT fragment lengths were detected in SJCRH30 *POLG* WT and Y955C using the aforementioned enzymes and the digoxigenin (DIG)-labeled ss-oligonucleotide H-strand probe targeted to *ND1*. A single high-molecular-weight (HMW) mtDNA band was observed for PvuII-digested DNA samples, which agrees well with previous results for the human cell line HepaRG (44). When *POLG* WT and Y955C whole-cell extracted (WCE) DNA samples were separately digested with BamHI plus NheI, a major band with a calculated molecular weight (MW) of 7.1 kb was observed in both, which is in good agreement with the expected length of the BamHI/NheI fragment containing the region for the *ND1* probe, 6.9 kb (Fig. 6, *A* and *C*). Interestingly, using the *ND1* probe, we detected a couple of minor intensity bands at ∼6.3 and 4.9 kb in the WT BamHI/NheI double digest (expected lengths of 6.5 and 4.4 kb, respectively). The minor intensity bands may represent replication intermediates resulting from stalling of the replisome during strand-displacement mtDNA. Stalling of Polγ during counterclockwise lagging-strand synthesis (nascent L-strand) at the predicted G-quadruplex (G4) located at 4229 to 4260 bp, followed by a strand break in the template H-strand, could result in the minor intensity BamHI/NheI digest band with the expected 6.5 kb length, Figure 6*B*. A previous study showed that mtDNA G4 forming sequences are associated with mtDNA deletion breakpoints suggesting a role for G4s in perturbation of mtDNA replication by fork stalling (45, 46). In addition, G4s are associated with nuclear genome instability and gene expression defects (47). G-quadruplexes are noncanonical secondary structures formed by planar stacking of four guanines called a G-tetrad, and two or more G-tetrads can stack to form a thermodynamically stable quadruplex (46, 47). Furthermore, mtDNA base pairs 4229 to 4260 localize to a previously identified region of replication pausing found in human tissue (brain, heart, skeletal muscle, placenta, kidney) and cell (HEK293T, HeLa, 143B, Jurkat) mtDNA (48).

The expected BamHI/NheI 4.4- (4.9 observed) kb fragment detected with *ND1* could be produced if the clockwise leading strand (nascent H-strand) Polγ stalled (or just finished H-strand synthesis) around O_H_ and an L-strand/template strand break occurs in this region. Replication initiation of nascent leading H-strand synthesis at O_H_ involves a complex hybrid G4 between mitochondrial RNA generated by mtDNA transcription and the nontemplate H-strand (4). We predict this hybrid structure can stall or signal the completion of Polγ nascent H-strand synthesis at O_H_ resulting in the production of the minor 4.4-kb species on BamHI/NheI digest when a template strand break occurs. Curiously, in Y955C compared with WT, the *ND1*-probed 4.4-kb band is diminished and the 6.5-kb band is not detectable. Finally, in agreement with an expected mtDNA XbaI cleavage product of 4.5 kb, harboring the complementary *ND1* probe sequence, a band with a calculated MW of 4.8 kb was detected in both cell types.

Next, an H-strand ss-DIG-labeled oligonucleotide complementary to the major arc *ND4* gene was used. A single HMW band was observed for PvuII-digested WT and Y955C DNA samples, which is in good agreement with what was observed with the *ND1* probe described above. When *POLG* WT and Y955C WCE DNA samples were separately double digested with BamHI and NheI and then probed with *ND4*, a major band with a calculated MW of 9.4 kb was observed in both samples and agreed with the expected length of the 9.7-kb BamHI/NheI fragment, Figure 6. In addition, using the *ND4* probe with BamHI/NheI digests, a faint band at 8.7 kb (expected MW 8.5 kb) was observed that could represent a truncated counterclockwise nascent L-strand fragment that lacks the NheI site due to initiation from O_L_ and a break in the template H-strand near this origin. Following XbaI digestion of WCE DNA samples, Southern blotting, and *ND4* probe hybridization, we detected a major band at 7.9 kb in both the cell types, which is in good agreement with the expected length of the ds-mtDNA 7.5 kb probed XbaI fragment. Also, two less intense bands were observed below the 7.9-kb band in both the cell lines, 6.8- (6.5 expected) and 4.8- (5.3 expected) kb bands, although the two bands were less prominent in Y955C. In the strand-displacement model of mtDNA replication, the 6.5-kb band can be rationalized by clockwise initiation of nascent H-strand mtDNA synthesis at O_H_ with a break in the template L-strand in this region and cutting by XbaI at position 10,257 in the mtDNA genome. On the other hand, the expected 5.3-kb band could represent a species initiated from counterclockwise mtDNA synthesis at O_L_ with Polγ stalling at the H-strand/template strand G4 located at position 15,545 to 15,516. In this scenario, the 5.3-kb band requires a single-strand break near the H-strand G4 sequence to be visualized upon XbaI digestion. We obtained similar results with ss-DIG-labeled oligonucleotide L-strand probes localizing to *ND1* and *ND4* (Fig. S2).

To further rule out the possibility that the minor bands detected with the *ND1* and *ND4* probes (bands 3, 4, 7, 9, and 10) resulted from mtDNA deletions, we used long-range PCR to screen for truncated deletion products. We did not detect significant deletions in the WT or Y955C cell lines. Overall, these data support that the minor intensity bands of lower-than-expected molecular weights predominantly result from single-strand breaks of replication intermediates initiated at O_H_ or O_L_ and replisome stalling events at G-quadruplexes.

### SJCRH30 *POLG* Y955C cells have fewer nucleoids compared with wildtype cells

**Figure 7:**
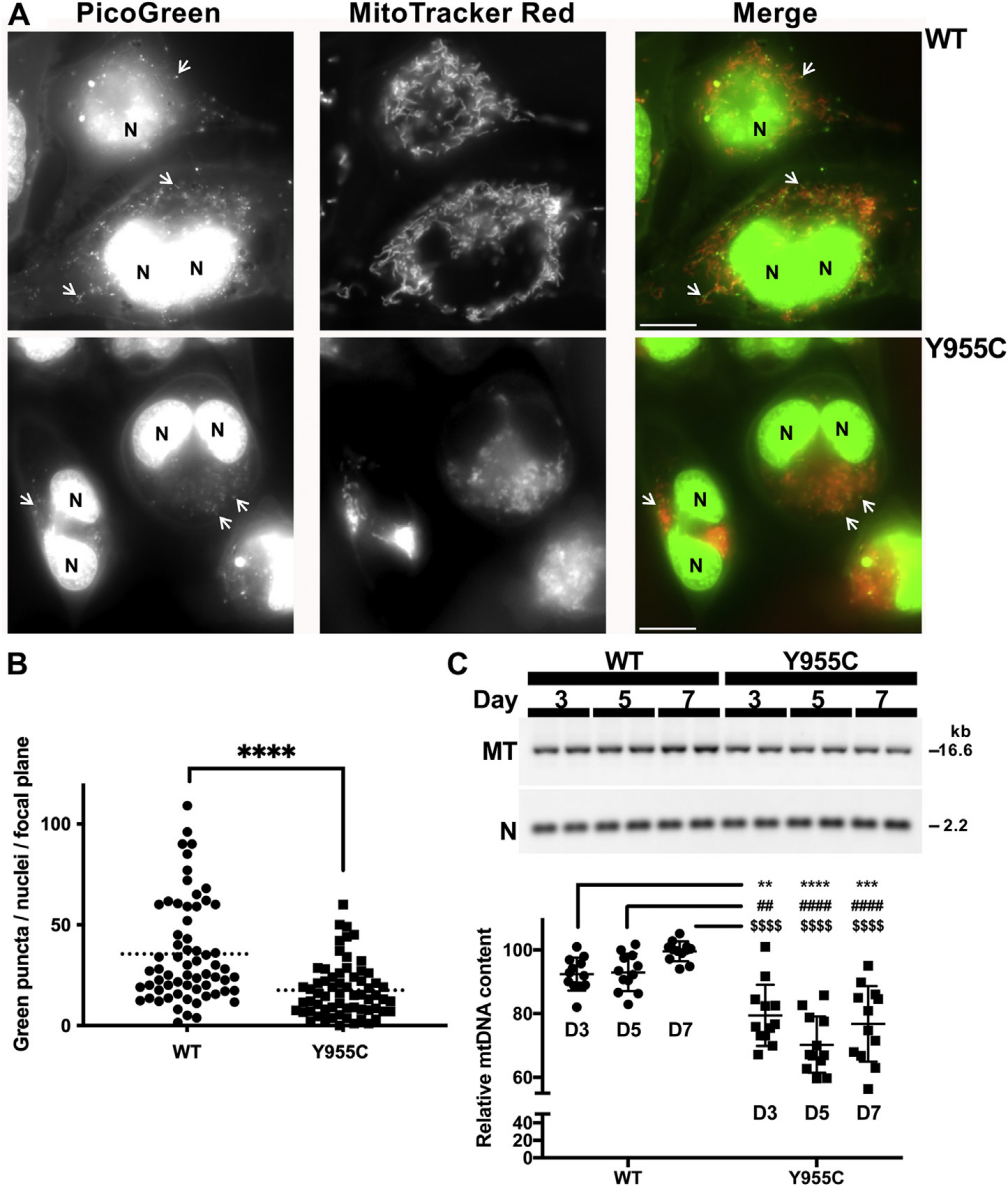
**SJCRH30 *POLG* Y955C cells contain less mtDNA and fewer mtDNA nucleoids than *POLG* wildtype cells.***A*, *POLG* wildtype (WT) and Y955C cells were separately dual stained with MitoTracker *Red* and *PicoGreen* dsDNA Reagent, and live-cell images were collected on a fluorescent microscope. Three P*OLG* WT and four Y955C *PicoGreen*-stained nuclei are labeled “N,” and three *PicoGreen*-stained mtDNA nucleoids are emphasized with white *arrows* in both cell types. For clarity, the *PicoGreen* (*green*) and MitoTracker *Red* (*red*) channels are shown separately in *grayscale* and together in color in the merged images. *A* representative image for each cell type is shown. Scale bars represent 10 μm. *B*, mitochondrial nucleoids from n = 64 total cells were counted for each *cell line* and from two different experiments (n = 32 cells for each) using different passages; ∗∗∗∗*p* < 0.0001. *C*, BamHI-digested whole-cell extracted DNA samples were analyzed *via* Southern blot and nonradioactive probe hybridization. The blots were simultaneously probed with the DIG-labeled 18S nDNA probe (N, *lower panels*; nucleotide positions 101–600) and the mtDNA-specific probe (MT, *upper panel*; nucleotide positions 168–606). Bands were quantitated using the open-source image-processing package Fiji, as described (44, 53). A representative blot is shown. On each blot, the average normalized band intensity values of WT mtDNA relative to nDNA on day 7 were set to 100%, and all other samples were compared with it. Statistical significance was determined by a two-way ANOVA, n = 12 (quadruplicates from three experiments using different passages, a total of six blots were analyzed, two per experiment); ∗∗∗∗, ####, and $$$$ *p*< 0.0001; ∗∗∗*p* < 0.001; ## and ∗∗*p* < 0.01.

PicoGreen is a simple and effective dsDNA-specific probe used to image the tightly packed mtDNA nucleoid in living cells (49, 50). These nucleoprotein complexes are visualized as cytoplasmic foci or puncta that colocalize with mitochondria using fluorescence microscopy (51). Live cell mitochondria are effectively detected using the MitoTracker Red fluorescent probe (51). PicoGreen was employed to label total cellular dsDNA (mtDNA + nDNA) with green fluorescence, and MitoTracker Red was used to label mitochondria. PicoGreen-stained mtDNA nucleoids (puncta) localizing to MitoTracker Red–stained mitochondria were counted in the *POLG* WT and mutant cells. Relative to focal planes containing SJCRH30 *POLG* WT cells, *POLG* Y955C cells have half the PicoGreen stained nucleoids (*p* < 0.0001), Figure 7, *A* and *B*.

Mitochondrial morphology was assessed in ≥111 cells of each type using the Mitochondrial Analyzer plugin for ImageJ/Fiji (52). Compared with *POLG* Y955C mutant cells, WT cells have 1.5- to 2-fold increases in mitochondrial area, counts, perimeter, branches, branch lengths, branch endpoints, and branch junctions, Table 1. This image analysis agrees with the 1.7-fold increase in mitochondrial protein level per cell detected in our WT mitochondrial extract compared with the Y955C mutant (see Isolation of mitochondria under Experimental procedures). In addition, an increased level of WT mitochondrial branch endpoints (where branches end without connecting to another branch) helps explain the notable WT tubular-shaped mitochondria compared with the more clustered appearance of the Y955C organelles, Figure 7*A*.

**Table 1:** Comparison of SJCRH30 *POLG* wildtype and Y955C mitochondria

Measurements normalized per cell^a^	SJCRH30 *POLG* wildtype^b^	SJCRH30 *POLG* Y955C^b^	Fold-change	P-value^c^ (fold-change)
Mitochondria (count)	54.2 ± 5.1	37.0 ± 3.7	1.5	< 0.002
Mitochondrial total area (μm^2^)	54.7 ± 4.9	33.9 ± 6.1	1.6	< 0.002
Total perimeter of mitochondria (μm)	257.0 ± 23.5	156.7 ± 17.8	1.6	< 0.0005
Mitochondrial branches	80.8 ± 7.6	50.1 ± 4.6	1.6	0.0005
Total mitochondrial branch lengths (μm)	86.2 ± 9.3	48.5 ± 10.7	1.8	< 0.002
Mitochondrial branch endpoints	109.9 ± 11.1	72.5 ± 5.6	1.5	< 0.001
Mitochondrial branch junctions	14.3 ± 1.9	7.2 ± 3.3	2.0	< 0.01

a Live-cell MitoTracker Red CMXRos staining, fluorescence microscopy, and imaging were carried out as described under Experimental procedures. Measurements were made using The Mitochondrial Analyzer plugin for ImageJ/Fiji and data are normalized to values per cell (see “MitoTracker Red CMXRos-stained mitochondria measurements”).

b Data are mean ± SD, n = 4 images with ≥ 111 total cells (two digital photographs from independent experiments performed on different days with different preparations of cells).

c *p*-Values < 0.05 were accepted as significantly different.

### The *POLG* Y955C mutation causes decreased mtDNA copy number

Compared with the parental *POLG* WT cells, the reduced number of *POLG* Y955C mtDNA nucleoids and stalled replication intermediates revealed by restriction endonuclease mapping supports that the mtDNA copy number is lower in the mutant. Therefore, to determine whether the *POLG* Y955C mutation causes mtDNA depletion, the mtDNA copy number was investigated using our relative copy number method (53). The WCE DNA samples were prepared from cells obtained on days 3, 5, and 7 post seeding as described under Experimental procedures. The levels of mtDNA in each lane of the blot were detected with the mtDNA-specific probe and were normalized to nDNA levels seen with the 18S probe in the same lane, Figure 7*C*. In the *POLG* WT cell line, we observed an increase in mtDNA copy number over the 7-day experiment; therefore, WT mtDNA on day 7 was normalized to 100%. On days 3, 5, and 7 of growth, the SJCRH30 WT mtDNA copy number values were 92.4 ± 5.1, 92.9 ± 5.9, and 100.0 ± 3.1%, respectively. At the same time points, the SJCRH30 *POLG* Y955C mutant mtDNA values were 79.5 ± 9.6, 70.3 ± 8.8, and 76.8 ± 11.9%, respectively. A two-way ANOVA was done to assess the interactions at different time points and between the two different genotypes (*POLG* WT and Y955C). Throughout the experiment, we did not observe significant changes in mtDNA copy number within each genotype/group; however, significant differences between WT and Y955C were detected at all the time points analyzed. When comparing mtDNA content between WT and Y955C on days 3, 5, and 7, Y955C mitochondrial genomes were reduced by 14.0%, 24.3%, and 23.2% relative to WT on the same day. Therefore, mtDNA content in Y955C is significantly reduced relative to the WT cell line during standard cell culture growth conditions. We suspect that the more severe Y955C mtDNA depletion detected with PicoGreen (∼50%) as compared with our Southern blotting technique (∼20%) is due to the specificity of PicoGreen for dsDNA over ssDNA as stated by the manufacturer (Thermo Fisher Scientific) and others (50).

### SJCRH30 *POLG* Y955C cells accumulate mtDNA replication intermediates

One-dimensional (1D) and two-dimensional (2D) agarose gel electrophoresis (AGE) are powerful tools used to analyze the numerous complex mtDNA genome topological structures that exist within a cell such as supercoiled, linear, relaxed circles, and various catenanes (40, 54, 55, 56). An elegant study by Kolesar *et al.*(40) demonstrated that, among different cell types and tissues derived from humans and mice, there exist similar major mtDNA topoisomers (*e.g.*, catenanes, relaxed circles, linear molecules), but the mtDNAs can be distributed differently, and additional structures can be seen depending on the cell type or tissue. We speculated that the *POLG* Y955C mutant cell line could harbor different distributions of mtDNA topoisomers relative to the WT. To investigate mtDNA topological structures, we separately isolated total cellular DNA samples (WCE DNA) from *POLG* WT and Y955C cells and subjected them to RNase A to remove RNA and reveal regions of ssDNA. Next, the WCE DNA samples were subjected to 1D- or 2D-AGE, Southern blotting, and nonradioactive probe hybridization to visualize mtDNA topoisomers.

**Figure 8:**
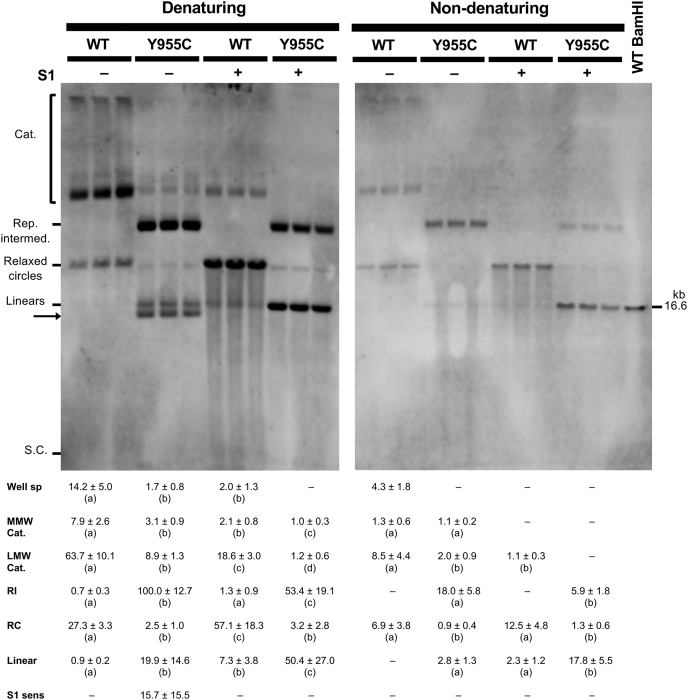
**The SJCRH30 *POLG* Y955C *cell line* harbors additional mtDNA topoisomers sensitive to S1 nuclease.** WCE DNA samples were treated with RNase A and digested with BglII to fragment nDNA (but not mtDNA). Where indicated, samples were treated with S1 nuclease (S1). Alkaline denaturation (Denaturing) before Southern blotting is necessary for hybridization of dsDNA with a single-stranded (ss) probe but can be omitted to assess potential single-stranded DNA species. For nondenaturing analysis (Non-denaturing), the gels were excluded from the denaturing solution step, but the other steps were performed. The blots were probed using the DIG-labeled mtDNA-specific probe (nucleotide positions 168–606). The untreated denatured samples (without S1 nuclease) contain major mtDNA topoisomers such as catenanes (Cat.), relaxed circles (RC), linears, and supercoiled (S.C.) molecules. Catenanes include HMW mtDNA well species (Well sp), mid-range-MW (MMW) catenanes, and low-MW (LMW) catenanes. Y955C cells accumulate a large amount of a replication intermediate (Rep. intermed. or RI) that localizes between the LMW catenanes and RCs, is sensitive to S1 nuclease, and is barely detectable in the WT. Under denaturing conditions, a unique species was seen below the linear mtDNAs in Y955C untreated samples and is sensitive to S1 nuclease digestion (S1 sens, highlighted with an *arrow* on the blot). A BamHI-digested WT sample was run in *parallel* as a control for linear ∼16.6-kb mtDNA. Data below the blots are mean values ± SD. Mean and SD values were calculated from n = 6 data points from two sets of blots, four in total (two denatured and two nondenatured, all photographed on the same image and probed with the same batch/concentration of probe, a representative blot for each is shown). The three replicates for each treatment shown are separate WCE DNA preparations from different passages of cells. The denatured and nondenatured blots were separately analyzed, and topoisomer differences among WT untreated, WT S1 nuclease treated, Y955C untreated, and Y955C S1 nuclease treated were determined (the *rows* of the table below the blots were compared). Three or more data sets per *row* were compared using a one-way ANOVA or a Welch’s ANOVA, while data sets of two (*e.g.*, nondenatures MMW catenanes) were analyzed using a *t* test. Identical lowercase letters within a *row* ((a) *versus* (a) or (b) *versus* (b)) indicate no significant difference in the mean level of topoisomers, while different letters represent statistically significant differences. *p* < 0.05 is considered significant, and all *p*-values were <0.031.

First, 1D-AGE was used to resolve HMW mtDNAs, and the species that formed discrete bands were quantitated. Catenanes and relaxed circular (RC) mtDNAs were the major topoisomer species observed in the parental *POLG* WT WCE DNA. Catenanes included HMW mtDNA structures found in the gel wells (well species), mid-range-molecular-weight (MMW) catenanes, and low-molecular-weight (LMW) catenanes, Figure 8. A smear of signal from the WT well species down to the MMW catenanes is indicative of additional HMW complex catenated species that exist *in vivo*. Two other minor species were quantitated, a barely detectable replication intermediate (RI) band and a faint linear mtDNA band (Fig. S3 shows overexposed replicate blots to highlight the minor species). We treated the WCE DNA samples with S1 nuclease, an ssDNA-specific nuclease, to determine whether the mtDNA structures contain ssDNA. Compared with untreated WT samples, a 7.1-fold reduction in well species and a decrease in the smear from the well down to the MMW catenanes were detected following S1 treatment. These observations are in agreement with Kolesar *et al.* (40). Also, following S1 treatment, WT MMW and LMW catenanes decreased 3.8- and 3.4-fold, respectively, while RC and linear mtDNA increased 2.1- and 8.1-fold, respectively. These results suggest that RC and linear species are released from the HMW molecules following S1 treatment. To support that the S1-sensitive species harbor ssDNA, we omitted the alkaline denaturation step before Southern transfer (nondenaturing conditions). Alkaline denaturation of DNA is necessary to hybridize a dsDNA with a single-stranded probe. Therefore, omission of the denaturation step can detect regions of ssDNA (40). Under nondenaturing conditions, we observed potential regions of ssDNA in the WT that include mtDNA well species, a weak smear of signal from the well species down to the MMW catenanes, MMW catenanes, LMW catenanes, and RC mtDNAs. After S1 treatment, the nondenatured WT well species, the smear of signal from the well species down to the MMW catenanes, and the MMW catenanes were undetectable; the LMW catenane species were barely detectable; and the RCs increased 1.8-fold relative to the untreated samples but were not significantly different from untreated. Linear nondenatured WT mtDNAs were undetectable in untreated samples but appeared after S1 nuclease treatment suggesting these molecules originated from the untreated nondenatured HMW species.

Strikingly, under alkaline denaturing conditions, the *POLG* Y955C mtDNA RI found between the LMW catenanes and the relaxed circles was the most abundant species on the blots and thus was set to 100% for normalization of the data, Figure 8. The Y955C RI was increased 143-fold relative to untreated WT under denaturing conditions. In addition to the RI, Y955C DNA extracts contained HMW well species, a weak smear of signal from the well species down to the MMW catenanes, MMW catenanes, LMW catenanes, and relaxed circles. The HMW well species, the MMW catenanes, the LMW catenanes, and the RCs were significantly less than those found in WT, being 8.4-, 2.6-, 7.2-, and 10.9-fold decreased, respectively. Also, relative to untreated WT WCE DNA samples, a 22-fold increase in Y955C linear mtDNAs was observed, and a unique band was detected under the linear band. Following S1 treatment of Y955C DNA extracts, the mtDNA well species, the smear from the well down to MMW catenanes, and the unique band under the linear mtDNA band were completely digested, suggesting these molecules harbor significant quantities of ss-mtDNA. In addition, the MMW and LMW catenated species were nearly completely digested, about half of the RI molecules were degraded, and the linear mtDNAs increased 2.5-fold. The decrease in the HMW well species, the smearing from the well down to MMW catenanes, and the increased abundance of linear mtDNAs agree with the results observed for WT; however, Y955C did not display an increase in the RC molecules following S1 digestion. Relative to WT and under nondenaturing conditions, we observed a significant signal for potential regions of ssDNA in the Y955C RI species and lower levels of LMW catenane and RC mtDNAs. Another difference from WT is that linear mtDNAs harboring potential regions of ssDNA were detected in untreated Y955C WCE DNA samples. Following S1 treatment, the nondenatured Y955C MMW and LMW catenane species were undetectable, RIs decreased 3.1-fold, and linear mtDNAs increased 6.4-fold. These results suggest that linear Y955C mtDNAs harboring regions of ssDNA were released from the MMW catenanes, LMW catenanes, and RIs following digestion with S1 nuclease. Surprisingly, the unique Y955C S1 nuclease-sensitive band found below the linear mtDNA band under denaturing conditions was not detected under nondenaturing conditions.

**Figure 9:**
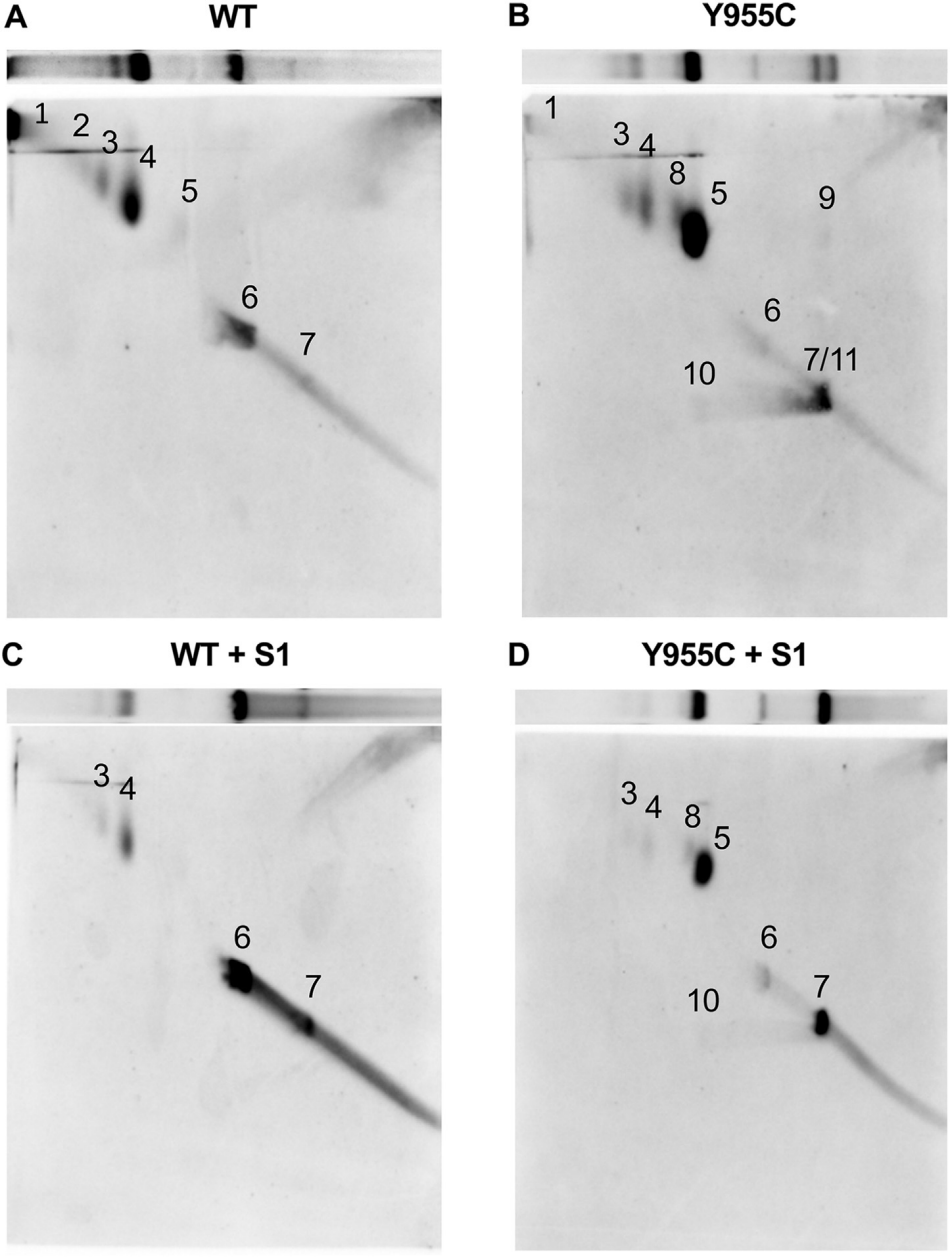
**Examination of mtDNA topoisomers by two-dimensional agarose gel electrophoresis (2D-AGE) reveals additional *POLG* Y955C-specific replication intermediates.***A*, *POLG* WT, *B*, *POLG* Y955C, *C*, *POLG* WT S1 nuclease treated, and *D*, *POLG* Y955C S1 nuclease treated WCE DNA samples. The WCE DNA samples were subjected to 2D-AGE, Southern blotting, and probe hybridization using the DIG-labeled mtDNA-specific probe (nucleotide positions 168–606). For comparison, above each 2D-AGE blot is a representative 1D *blot lane* in the orientation needed for the 2D separation. We identified 11 different mtDNA topoisomers in the two cell types and six are shared between WT and Y955C. Numbers from 1 to 11 are *1.* mtDNA well species, *2.* High-molecular-weight (HMW) catenanes, *3.* mid-range-MW (MMW) catenanes, *4. Low*-MW (LMW) catenanes, *5.* a replication intermediate (RI) found in Y955C and WT, *6.* relaxed circles (RC), *7.* linear mtDNAs, *8.* a minor Y955C-specific RI (first minor RI), *9.* a Y955C-specific S1-sensitive species, *10.* a second minor Y955C-specific RI (second minor RI), and *11.* a second Y955C-specific S1-sensitive species running near the linear band (second S1 sensitive species). See Fig. S4 for another set of example blots.

To further investigate the differences observed between *POLG* WT and Y955C mtDNAs on 1D-AGE, we used 2D-AGE as an additional method to examine mtDNA topoisomers. First, WCE DNA was separated by 1D-AGE as described above, and we cut lanes containing DNA samples of interest out of the gels. Second, individual lanes were separately caste into another gel with identical agarose concentration but now containing ethidium bromide (EtBr). EtBr was added to the running buffer to enhance the rigidity of DNA and exaggerate the separation of mtDNAs with differences in extended shape. In this scenario, mtDNA RIs move more slowly than linear mtDNAs (57). Also, EtBr induces further supercoiling in covalently closed or topologically constrained mtDNAs (40). The six SJCRH30 WT mtDNA species identified in 1D-AGE were seen by 2D-AGE, plus an additional catenane band above the MMW catenane band was resolved: 1. HMW mtDNA well species, 2. HMW catenanes, 3. MMW catenanes, 4. LMW catenanes, 5. a barely detectable RI, 6. RC, and 7. linear mtDNAs, Figure 9*A*. We treated the WCE DNA sample with S1 nuclease to examine mtDNA structures with ssDNA. Following treatment of DNA extracts with S1 nuclease, the HMW mtDNA structures in the well, the HMW catenanes, and the RI were undetectable, while the MMW and LMW catenanes decreased in abundance but were still detectable and RCs and linears increased, Figure 9*C*.

Compared with *POLG* WT cell mtDNAs, 2D-AGE revealed four new minor Y955C-specific structures. The new structures include *1.* a slower-moving, less abundant Y955C-specific RI (first minor RI; band 8), *2.* a faint Y955C-specific S1-sensitive species (band 9), *3.* a fast-moving second RI (second minor RI; band 10), and *4.* a second faint Y955C-specific LMW S1-sensitive species that migrated near the linear band (band 11), Figures 9*B* and S4. In addition, a subtle horizontal range (smear) of Y955C molecules spanning the region between the second minor RI (band 10) and the linear band (band 7) was revealed. The HMW catenane band 2 seen in WT cells was undetectable in Y955C, and the well species (band 1) was weak. Following S1 nuclease treatment, the well species and the S1-sensitive species (bands 9 and 11) were undetectable on the blot, the RC band 6 was about the same as the untreated sample, the linear mtDNA band 7 became sharper, and the other bands (3–5, 8, and 10) had decreased intensities.

### SJCRH30 *POLG* Y955C DNA extracts lack detectable four-way mtDNA junctions

**Figure 10:**
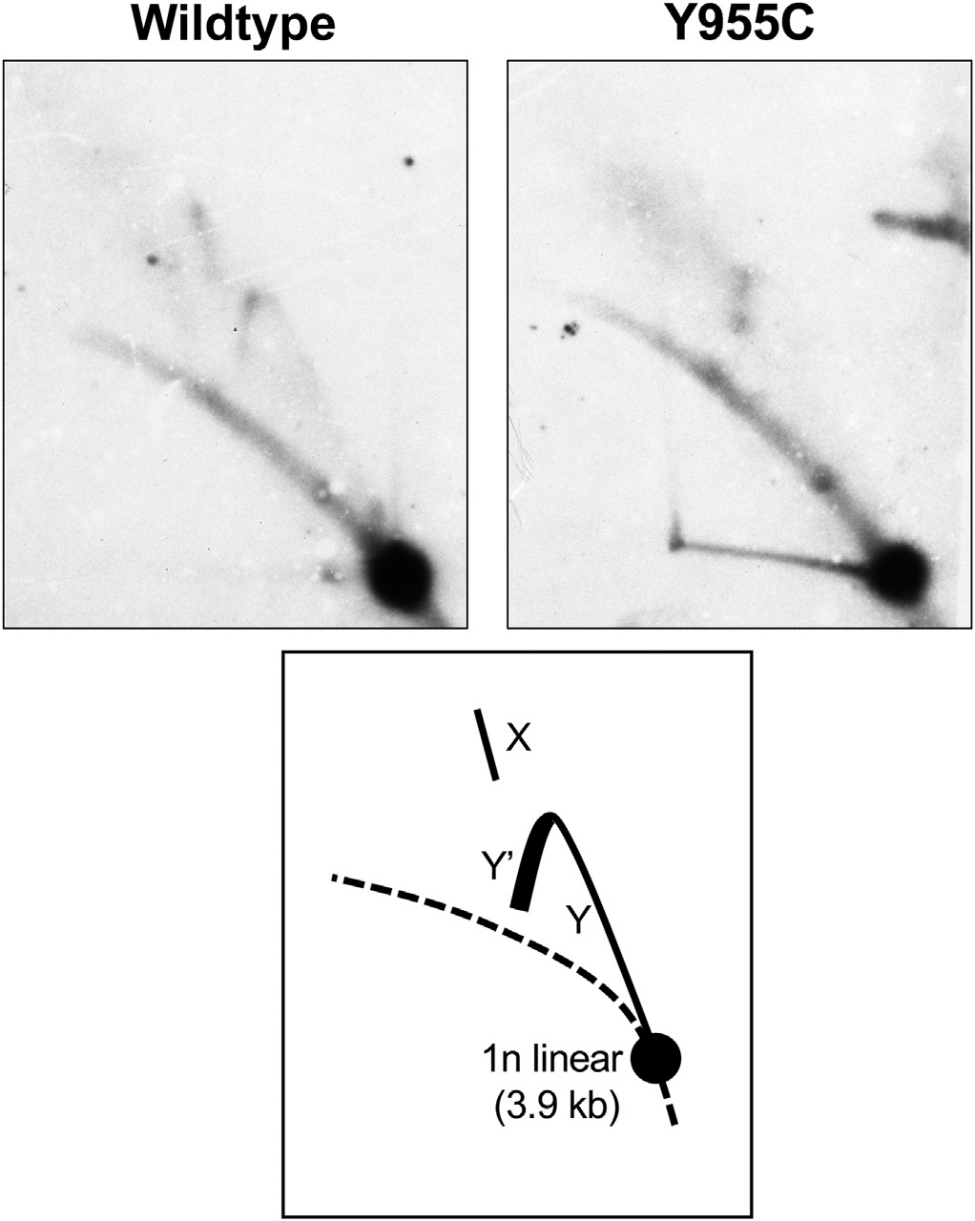
***POLG* Y955C mtDNA lacks detectable four-way DNA junctions (*i.e.*, hemicatenanes) and has a reduced replication fork signal.** WCE DNA was digested with HincII, subjected to 2DNAGE and Southern blotting and probed with a mtDNA-specific probe (positions 37–611). The mtDNA probe is specific to an ∼3.9-kb subgenomic mtDNA HincII restriction fragment harboring the heavy strand origin of replication (O_H_). The schematic below the wildtype and Y955C blots emphasize the 1n, 3.9-kb nonreplicating HincII fragment (*large black circle*); the ascending and descending parts of the Y arc (mtDNA replication fork) represented as Y and Y′, respectively; and X-form molecules (X), dimeric fragments joined by four-way junctions.

Our initial mtDNA restriction endonuclease mapping suggested that some WT RIs involve L/template strand breaks near O_H_ and that Y955C cells have reduced amounts of these molecules, indicating problems with mtDNA maintenance at O_H_, Figure 6. The end of mtDNA replication has been demonstrated to occur *via* a hemicatenane formed at O_H_, and topoisomerase 3α is essential for resolving this structure (39). In comparison with SJCRH30 *POLG* WT, Y955C cells suffer from depletion of monomeric RCs (Fig. 8), which according to the vertebrate strand displacement model, are generated following decatenation (58). Therefore, we hypothesized that following completion of mtDNA synthesis topoisomerase 3α decatenation of daughter mtDNAs may be altered in *POLG* Y955C cells. To dissect this further, we used two-dimensional neutral agarose gel electrophoresis (2DNAGE) and Southern blotting to visualize X-form mtDNA structures that are expected to result from hemicatenated molecules generated at O_H_. Indeed, in comparison with *POLG* WT, X-form dimeric fragments joined by four-way junctions (*i.e.*, hemicatenanes) were undetectable in Y955C and the ascending mtDNA replication fork arc (Y-arc) was very weak supporting our hypothesis that decatenation at O_H_ is altered in *POLG* Y955C, Figure 10. Also, a strong descending Y-arc signal combined with depleted X-forms is seen in other cases of mtDNA replication stalling, including ddC treatment, TFAM overexpression, or expression of catalytically defective Polγ or Twinkle. An interpretation is that the slow progression of replication causes an accumulation of Y-forms while the replication termination intermediates are depleted due to their constant resolution (54, 59, 60, 61).

### SJCRH30 *POLG* Y955C has increased sensitivity to 2′-3′-dideoxycytidine

**Figure 11:**
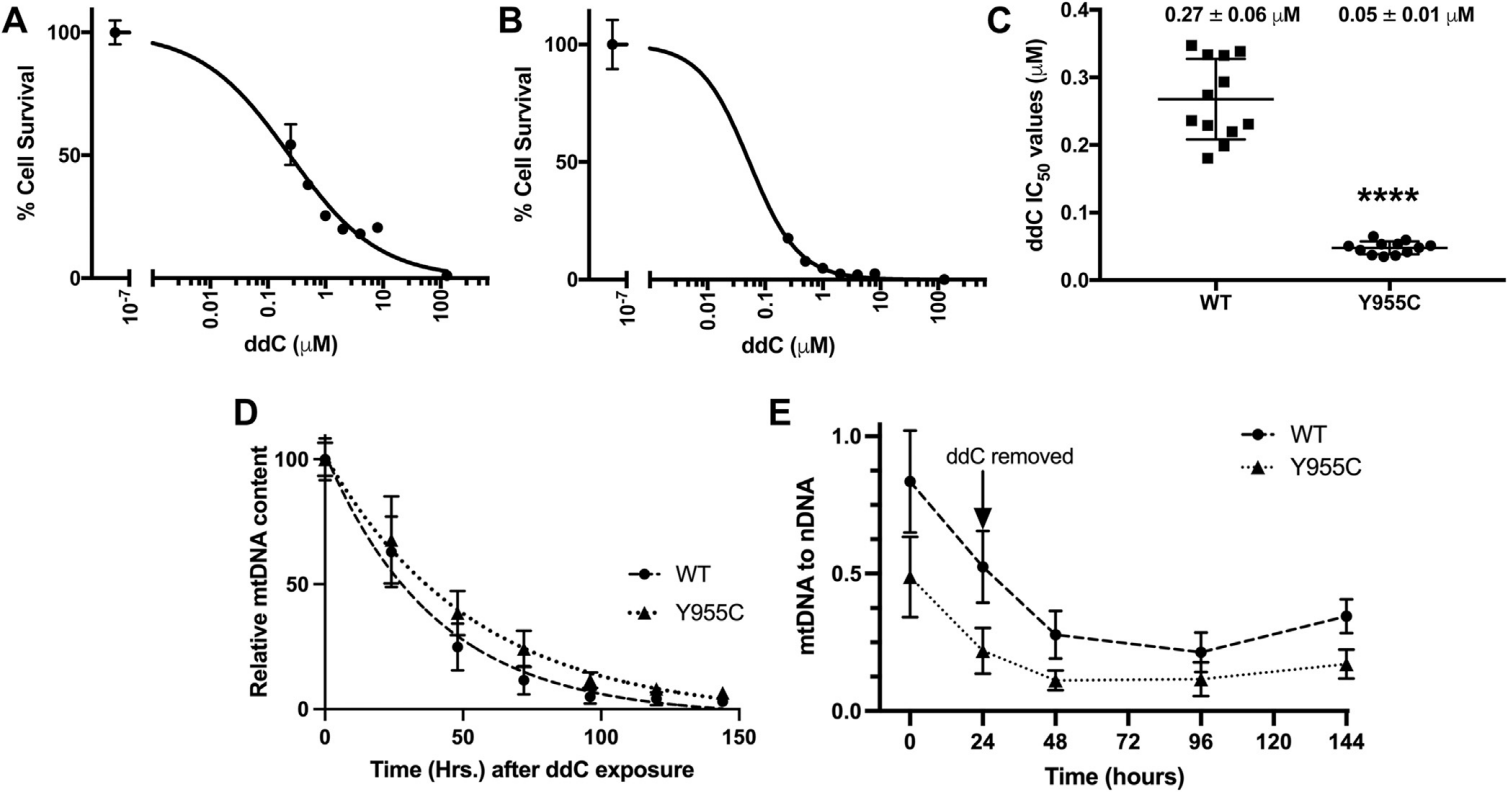
**SJCRH30 *POLG* Y955C cells have increased sensitivity to ddC.** In *A*, wildtype (WT) passage 16 and *B*, Y955C passage 12 experiments, cells were exposed to 128, 8, 4, 2, 1, 0.5, 0.25, and 0 μM ddC for 9 days and mean percent survival values (n = 4) and SDs are reported for each representative IC_50_ curve. *C*, IC_50_ values were calculated in quadruplicate from three independent experiments (n = 12) utilizing WT at passages P10, 12, and 16, and Y955C at 6, 8, and 12. IC_50_ values were calculated using the least-squares fit of inhibitor concentration *versus* normalized response in Graph Pad Prism, and mean values are reported in micromolar (μM) with error as SD. *D*, the relative rate of mtDNA depletion is similar in WT and Y955C cells exposed to 1 μM ddC. The rate of mtDNA depletion in WT and Y955C was determined by preparing WCE DNA samples from cells separately exposed to ddC followed by BamHI digestion, Southern blotting, and dual DIG-labeled probe detection as outlined in Figure 7. On each blot and for each cell type, the day 0 to 6 samples were loaded in duplicate, the average normalized band intensity values of mtDNA relative to nDNA on day 0 samples were set to 100%, and all other samples were compared with it. The experiments were repeated in triplicate (n = 12, quadruplicates from three experiments using different passages, a total of six blots were analyzed for each cell type, two blots per experiment). The average half-life values for mtDNA depletion were WT 29.6 ± 10.5 h and Y955C 40.0 ± 15.4 h, *p* = 0.064 by Student’s *t* test; errors reported are SD. The half-life values were calculated using the least-squares fitting method of the one-phase decay model in Graph Pad Prism. *E*, Recovery of mtDNA copy number following 24 h of 1 μM ddC treatment. The mtDNA genome copy number was measured using the dual probe method described in *D*, and the mtDNA to nDNA values (n = 8) are reported for two different experiments (quadruplicates for each). For each cell type, four Southern blots were analyzed.

Further complicating mitochondrial dysfunction, genetic mutations encoding variants of DNA polymerase subunits, which localize to the mitochondrion, could predispose patients to mitochondrial toxicity, *e.g.*, p140 (R964C, R953C, and E1143D/G) and PrimPol D114N (62, 63, 64, 65, 66, 67). For example, in two lymphoblastoid cell lines harboring *POLG* p.R964C, 10 μM of the nucleoside reverse transcriptase inhibitor stavudine (2′,3′-didehydro-2′,3′-dideoxythymidine) reduced mtDNA levels in the mutant cell lines but not in WT lymphoblastoid cell lines. Furthermore, compared with recombinant WT p140, p140 R964C had only 14% DNA polymerase activity (62). Therefore, if the *POLG* Y955C mutation enhances mitochondrial toxicity, then the mutant cell line can serve as a sensitive system to identify unidentified or hard-to-detect mitochondrial stressors. To test whether Y955C has enhanced mitochondrial toxicity, we exposed SJCRH30 *POLG* WT and Y955C cells to a known mitochondrial toxicant, the nucleoside reverse transcriptase inhibitor ddC. Using various human cell lines grown in tissue culture, ddC has been shown to induce mtDNA replication stress and, consequently, mtDNA depletion (49, 53, 68, 69, 70, 71, 72, 73); therefore, we expect Y955C cells will be more sensitive to ddC. The half-maximal inhibitory concentration (IC_50_) value or concentration of ddC that reduces the number of viable treated cells by 50% relative to untreated control cells was determined. Indeed, *POLG* Y955C cells are more sensitive to ddC, as indicated by a 5.4-fold reduction in the mutant IC_50_ value relative to WT, Figure 11, *A*–*C*. However, after exposing cells to 1 μM ddC and monitoring mtDNA depletion over 6 days, the depletion rates were not significantly different in the two cell types, Figure 11*D*. Also, we monitored mtDNA replication following a 24-h treatment of cells with ddC. Interestingly, when the WT and mutant cells were seeded at a high density, mtDNA copy number continued to decline after the removal of ddC. The mtDNA genome levels slightly increased from 96 to 144 h (3 to 5 days post treatment, respectively) in both cell types, Figure 11*E*. At 144 h, the WT and Y955C mtDNA copy numbers were 1.6- and 1.5-fold higher than at 96 h, respectively. However, only the 1.6-fold increase in WT mtDNA was significantly increased (*p* < 0.002 WT, *p* = 0.07 Y955C), suggesting recovery of mutant mtDNA replication is impaired.

## Discussion

Greater than 400 genes (mtDNA + nDNA) are currently linked to mitochondrial disease, and many additional disorders and nongenetic factors are associated with SMD (74). In addition, greater than 300 pathogenic mutations localize to the nuclear *POLG* gene alone and are associated with different types of mitochondrial disease, including mtDNA depletion and deletion disorders (75, 76). To better understand how a *POLG* mutation affects mitochondrial function at the cellular and molecular levels, we constructed a human cell line model harboring a knock-in of the most common autosomal dominant *POLG* mutation, Y955C. Another goal was to understand if the mutation confers increased sensitivity to the well-known mitochondrial stressor, ddC.

In agreement with previous reports of reduced mtDNA content in *POLG* Y955C adPEO patient skeletal muscle samples (10), patient-derived fibroblast culture (11), and model systems harboring the orthologous mutation including yeast (20), *Drosophila* (10), and mouse (22) we observed decreased mtDNA content in SJCRH30 *POLG* Y955C. However, in agreement with other studies discussed above, we only observed an ∼14% to 24% decrease in Y955C mtDNA copy number relative to WT over 7 days, Figure 7. These findings suggest that sufficient expression of the mutant’s WT *POLG* locus provides a necessary stoichiometry of p140 catalytic subunits *in vivo* or another DNA polymerase localizing to the mitochondrion assists the WT enzyme in replicating the mtDNA genome. Long-range PCR and restriction endonuclease digestion of total cellular DNA extracts using enzymes that cut once in the mtDNA genome (*i.e.*, PvuII or BamHI) followed by Southern blotting and probe hybridization did not reveal mtDNA deletion products shorter than 1n or 16,569 kb (Figs. 6 and 7). Similarly, in the orthologous fly models, mtDNA deletions did not present in the heterozygous flies or the homozygous larvae, and deletions were not reported in the yeast model, the mouse model, or in patient-derived cultured fibroblasts (10, 11, 20). As skeletal muscle mtDNA deletions can be present at low levels requiring long-range PCR to detect them (11), we suspect that differences in postmitotic skeletal muscle tissue *versus* mitotic tissue/cell mtDNA maintenance could account for the observed discrepancies in deletions. In addition, low levels of mtDNA deletions in actively dividing cell cultures have been speculated to result from purifying selection due to mitophagy (76). Future studies comparing mtDNA maintenance in cycling *versus* noncycling cells will address this possibility.

*In vitro* biochemical analysis has demonstrated that the p140 Y955C variant catalytic subunit decreases specificity and incorporation of dGTP, TTP, and dATP by ∼30-, 110-, and 1300-fold, respectively (77). Also, Y955C was shown to reduce the fidelity of Polγ nucleotide incorporation by ∼6 to 120-fold (77). Another study analyzed the mutation frequency of Y955C by creating exonuclease-deficient (exo^–^) variants of WT and Y955C. The study showed that the exo^–^ Y955C increased the mutation frequency around 42-fold relative to WT p140 while the exo+ Y955C variant showed a 2-fold increase in its mutation rate (19). In agreement with previous biochemical reports showing that p140 Y955C is dominant-negative *in vitro* (10, 13), we detected the accumulation of a large quantity of aberrant mtDNA replication intermediates in our mutant cell line that likely represents stalling of replisomes containing the p140 Y955C variant. Denaturing 1D-AGE revealed *POLG* Y955C mtDNA replication intermediates, including a major RI that localizes between LMW catenanes and RCs, plus a Y955C-specific S1-sensitive band localizing under the linear band. Using linear regression of the log10 base pair values of Lambda DNA/HindIII Marker 2 fragments loaded onto the same gel *versus* distance traveled in millimeters, and assuming the Y955C-specific S1-sensitive species is a linear molecule, its apparent MW is 15.9 ± 0.4 kb (based on n = 6 measurements from two experiments performed in triplicate). This size is much larger than the previously reported ∼11-kb subgenomic fragment seen in the MGME1 knockout mouse, the *POLG* mtDNA mutator mouse, and an MGME1-null patient fibroblast (78, 79) and suggests the band is the result of a Y955C-specific defect.

**Figure 12:**
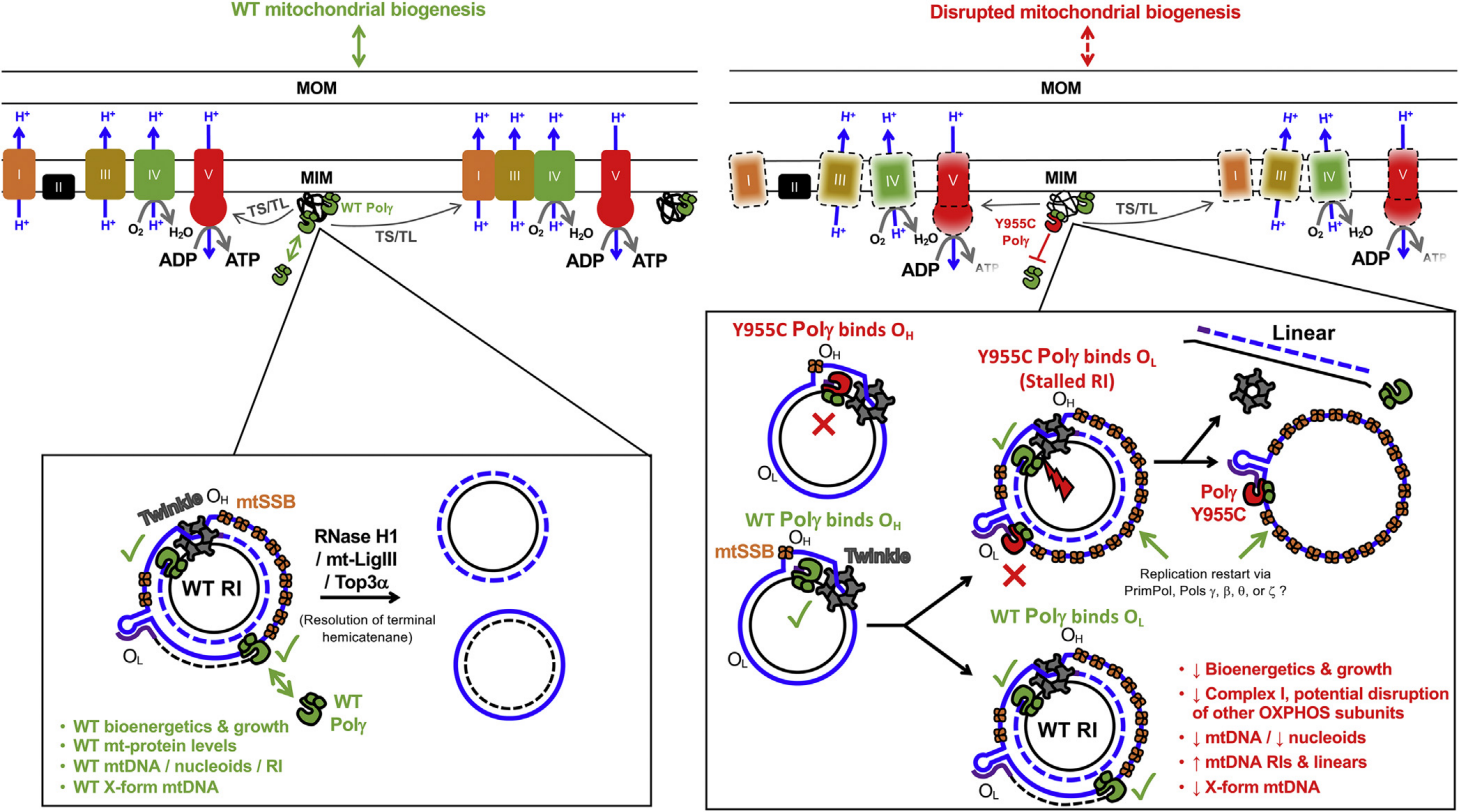
**A model to explain the cellular deficits resulting from the *POLG* Y955C mutation.***Left-hand side*, model of wildtype (WT) mitochondrial functions. The OXPHOS complex I, II, III, IV, and V components are labeled accordingly. The inset below shows the strand displacement model's continuous leading and lagging strand synthesis. *Right-hand side*, model of how the dominant-negative *POLG* Y955C mutation causes mitochondrial dysfunction. The inset highlights how Polγ Y955C could block mtDNA synthesis at the heavy strand origin of replication (O_H_) or the *light strand* origin of replication (O_L_) and generates increased levels of mtDNA linear molecules and stalled replication intermediates (RI). The red X represents a block in mtDNA replication, while WT replication is indicated with a *green checkmark*. *Solid lines* represent parental strands, while *dashed lines* represent newly synthesized mtDNA. The heavy (H) strands are *colored blue*, and the *light* (L) strands are *black*. The small *purple lines* represent RNA primers. The *red lightning* bolt represents a mtDNA L-strand break. WT Polγ, the replicative mtDNA polymerase gamma (*green*); Y955C Polγ (*red* catalytic subunit with *green* processivity subunits); Twinkle, Twinkle mtDNA helicase (*gray*); mtSSB, mitochondrial single-stranded DNA binding protein (*orange*); RNase H1, ribonuclease H1; mt-LigIII, mitochondrial DNA ligase III; Top3α, DNA topoisomerase 3 alpha; MOM, mitochondrial outer membrane; MIM, mitochondrial inner membrane; TS, mitochondrial-specific transcription; TL, mitochondrial-specific translation. DNA polymerases beta, theta, and zeta are labeled Pols β, θ, and ζ.

The major RI appears to harbor a mixture of ss- and ds-mtDNA molecules as the band intensities decreased following S1 nuclease digestion on both denaturing and nondenaturing analyses (Fig. 8). Based on the strand-displacement model, we hypothesize these molecules could consist of structures where leading nascent H-strand synthesis was initiated using a Polγ WT replisome at O_H_. In this scenario, following the Polγ WT replisome’s initiation at O_H_, lagging-strand synthesis is initiated by a dominant-negative Y955C replisome that stalls at a region near O_L_ leaving the parental H-strand displaced as ss-mtDNA, Figure 12. A previous *in vitro* order-of-addition biochemical experiment supports that when initially added to a primer-template reaction Polγ Y955C can block the access of Polγ WT to synthesize DNA (13). Interestingly, in the same study, when Polγ WT was preincubated, and then Polγ Y955C was added, DNA synthesis products were lost over time, supporting the idea that Polγ Y955C chews back the primer strand in a dominant-negative fashion using its exo+ activity. Furthermore, the authors made the interesting observation that, in 1 μM dNTPs, Polγ Y955C has a specific defect in dATP incorporation, and DNA synthesis terminates just before dATP incorporation (13). *In vivo*, this is likely a relevant problem for *POLG* Y955C patients as 52 different strand-specific sequences harboring stretches of five or more poly-thymidine (poly-T) bases (≥TTTTT) occur in the NC_012920 mtDNA reference sequence and likely cause the variant enzyme to stall. Forty-one of these poly-T stretches localize on the H-strand. In support of the idea that the Y955C variant replisome stalls near O_L_, forming ss-mtDNA in the major RI, the mtDNA reference sequence contains a five-base poly-T tract at positions 5834 to 5830 in the H-strand (located near the 5′-ends of the defined nascent L-strands near O_L_) (80).

The Y955C RCs likely represent ds-mtDNA molecules that completed replication as the signal on the blot was not significantly different following S1 nuclease digestion (Fig. 8). Assuming equal expression of both the WT and Y955C alleles, we expect that half of the Y955C replisomes will stall on mtDNA molecules at O_H_ while the other half consisting of WT replisomes will load at O_H_ and initiate mtDNA replication, Figure 12 (see the right-hand-side inset). Eventually, the Y955C variant replisomes will likely dissociate from O_H_, allowing another polymerase to load at the D-loop. Following binding of the WT to O_H_, there will be a chance for a WT or Y955C replisome to bind O_L_. Under denaturing conditions, Y955C linear mtDNAs were significantly increased relative to WT. Also, when Y955C samples were analyzed under nondenaturing conditions to detect ss-mtDNA, a faint linear band was detected, which likely represents broken ends from stalled replication forks hybridizing with the noncoding control region probe that includes O_H_. Therefore, we hypothesize that most Y955C linear molecules result from a stalled dominant-negative Y955C replisome at O_L_ and a stalled leading strand WT replisome at O_H_ followed by a template L-strand break.

The Y955C major RI band and S1-sensitive band (below the linears) on 1D-AGE were further resolved by 2D-AGE. In the region of the major RI, two different bands were detected. First, a slightly slower-moving minor Y955C-specific RI that migrated close to the major RI was resolved in the second dimension and was somewhat resistant to S1 nuclease, suggesting a dsDNA component to the major RI (see band 8 in Fig. 9, *B* and *D*). Second, a fast-moving minor RI was revealed (band 10) that was also somewhat resistant to S1 nuclease. Also, a slightly S1 nuclease–resistant Y955C-specific smear of molecules was present from the minor RI band 10 down to the linear band 7. These mtDNA molecules likely harbor dsDNA and could represent transactions between the disease variant Polγ Y955C and Polγ WT. Alternatively, Y955C-specific ds-mtDNA replication intermediates could represent the displacement or dissociation of Polγ Y955C from the mtDNA followed by compensatory synthesis by an alternative DNA polymerase that localizes to the mitochondrion, *e.g.*, Polβ, Polθ, Polζ, or PrimPol (81, 82, 83, 84, 85).

The Y955C-specific S1-sensitive species located below the linear mtDNA band in the first dimension was resolved into two species on 2D-AGE, a slower-moving S1-sensitive species (band 9) and a faster-moving S1-sensitive species that migrates close to the linear ds-mtDNA (band 11), Figure 9. As this S1-sensitive species could not be detected under nondenaturing conditions, it could be composed of mostly dsDNA with small sections of ssDNA that do not anneal to the noncoding control region probe. Interestingly, the Y955C-specific band 11 (Fig. 9) migrates close to the linear ds-mtDNA band 7, suggesting it could be topologically similar but contains ssDNA gaps and thus is sensitive to S1 nuclease and migrates slightly faster than linears. Perhaps this S1-sensitive band represents a unique replication restart product formed on the RI downstream of the stalled O_L_ Y955C replisome. In this scenario, following a primase reaction, WT Polγ, PrimPol, β, ζ, or θ could replicate a stretch or stretches of nascent L-strands. A restart product forming downstream of O_L_ and terminating around the 2GQH G4 followed by breakages of the H-strand could produce an ∼15-kb band, which is close to the calculated MW of 15.9 kb described above. Also, if a Polγ Y955C is bound to a restarted nascent L-strand, a poly-T sequence in the H-strand could allow the enzyme to backtrack using its exo+ to create small S1 nuclease–sensitive single-stranded gaps as previously proposed (13).

Another interesting observation was that we did not detect *POLG* Y955C termination intermediates (X-form dimeric fragments in the noncoding control region) by 2DNAGE. This is consistent with replication stalling or slowing down, which manifests as an increase in mtDNA replication intermediates (Figs. 8 and 9) and a strong descending Y-arc (Fig. 10), resulting in delayed completion of replication and, therefore, depletion of the termination intermediates. Our initial restriction mapping suggested that *POLG* Y955C replication intermediates were different from *POLG* WT; however, the mutant still contained fragments that suggested strand breaks can occur in the L-strand near O_H_ (Fig. 6 bands 4 and 9). This type of break around the completion of nascent H-strand synthesis could cause the daughter mtDNAs to fall apart without forming a four-way junction (hemicatenane). In heart tissue, the catenated mtDNA network is proposed to be maintained by the replicating mtDNA replisome (40, 56). Perhaps in SJCRH30 cells, the LMW catenanes, MMW catenanes, HMW catenanes, and HMW well species also represent mtDNA networks maintained by the replisome, and L-strand breaks contribute to the decreased abundance of these molecules in *POLG* Y955C relative to WT (Figs. 8 and 9).

In addition to the mtDNA maintenance defects described above, the *POLG* Y955C mutation caused a slow-growth phenotype compared with the WT. To better understand the physiological aspects of the mutation, we performed detailed bioenergetic analyses. When Y955C cells were grown in a glucose medium, we found that they suffered ∼20% decreases in several mitochondrial bioenergetic parameters. In addition, during all stages of the Mito Stress test, we observed enhanced *POLG* Y955C ECAR relative to WT consistent with upregulation of glycolysis in a myoblast cell (lower panel of Fig. 3*C*). ECAR Stress tests revealed that Y955C cells had reduced basal ECAR relative to WT in the absence of glucose. However, *POLG* Y955C cells displayed enhanced dependence on the glycolytic pathway as glucose-stimulated ECAR, and ECAR capacity was increased. The increase in both parameters comes at the cost of reducing the glycolytic reserve. As the glycolytic reserve is an estimate of the unused glycolytic capability that could be utilized by the cell if ATP demand was increased, we speculated that Y955C cells will have enhanced sensitivity to stressors such as environmental toxicants.

Finally, when *POLG* Y955C cells were grown in a galactose medium to obligate mitochondrial OXPHOS rather than glycolysis, the mitochondrial bioenergetic deficiencies in the mutant became more apparent (Fig. 4). Our results support using *POLG* Y955C cells grown in a galactose medium when analyzing the mitochondrial effects of future toxicants. At the molecular level, we showed that Y955C OXPHOS is likely affected by decreased expression of complex I NDUFB8 and ND3 subunits (Fig. 5). These results agree with a study demonstrating reduced OXPHOS complex I and IV protein levels in postmortem substantia nigra neurons from patients with *POLG*-related disease (36) and precisely an autopsy-derived sample of pigment neurons from substantia nigra obtained from a Y955C patient. Respiratory chain immunoreactivity showed that the Y955C patient neurons had reduced complex I and intense positive complex II, and complex III and IV amounts were like controls (86). Similarly, we did not detect changes in levels of Y955C complex II (SDHB), III (UQCRC2), IV (COX2), or V (ATP5A). Furthermore, cytochrome oxidase–deficient fibers have been reported associated with Y955C patients (10, 76), suggesting that reduced expression of mtDNA-encoded COX 1, 2, or 3 subunits could destabilize complex IV, the respirasome, and OXPHOS supercomplexes. Although we did not detect changes in Y955C COX2 levels, the altered mtDNA replication in the mutant may affect the expression of the remaining polypeptide genes leading to destabilization of the OXPHOS machinery and disruption of mitochondrial biogenesis (Fig. 12).

Human mitochondrial dysfunction is further complicated by evidence that genetic mutations can enhance mitochondrial toxicity suggesting there are unidentified environmental and pharmacological stressors that cause mitochondrial toxicity in individuals harboring mutant alleles. For example, a recent report using the nematode *Caenorhabditis elegans* showed that the sublethal concentrations of silver nanoparticles, which are extensively used in consumer products and biomedical applications, alter the development, behavior, and mitochondrial function in worms that harbor genetic defects in mitochondrial dynamics (87). Furthermore, a recent study demonstrated that exposure to ddC induces mtDNA stress leading to activation of the STING1/TMEM173-mediated DNA sensing pathway and autophagy-dependent ferroptotic cell death (88). To test whether *POLG* Y955C cells have enhanced sensitivity to a mitochondrial stressor, we exposed cells to various concentrations of the mitochondrial genotoxic agent and anti-retroviral ddC, and IC_50_ values were determined. The Y955C mutant displayed a >5-fold decrease in IC_50_ relative to WT, supporting that the mutant is more sensitive to mitochondrial toxicants. However, the rates of mtDNA depletion induced by ddC were not different between the two cell types; Figure 11*D*. Southern blotting served as a powerful tool to characterize and identify the human degradosome, the mitochondrial machinery that degrades mtDNA (89). In the degradosome model, the p140 catalytic subunit of Polγ harboring the 3′-5′ exonuclease activity, the 5′-3′ mtDNA Twinkle helicase, and the 5′-3′ MGME1 exonuclease work in concert to quickly degrade linear mtDNA. As the p140 Y955C catalytic subunit exo+ is not affected by the Y955C substitution, it is not surprising that mtDNA genome degradation in the mutant and WT cells are similar when the replisomes are stalled in the presence of 2′-3′-dideoxycytidine monophosphate. Also, after 24-h ddC exposures, we observed significant recovery of WT but not mutant cell mtDNA copy number following 5 days of growth in a recovery medium (Fig. 11*E*). Recent reports have demonstrated the usefulness of using human cell lines to measure the mitochondrial safety of antiviral drugs such as molnupiravir, remdesivir, and others (90, 91, 92, 93, 94). While we have characterized the differential effects of a single stressor on Y955C and WT cells in this work, other antivirals, drugs, and environmental toxicants should be investigated in the future to determine potential *POLG* Y955C–toxicant interactions.

In summary, we engineered a cell line model of a heterozygous *POLG* Y955C mitochondrial disease mutation in the SJCRH30 cell line. We showed that cellular mtDNA maintenance, complex I subunit expression, bioenergetics, mitochondrial morphology, and growth are altered due to the mutation’s presence. In addition, we demonstrated that the Y955C mutation causes an increase in sensitivity to ddC. In future studies, the Y955C cell line should prove helpful for identifying other mitochondrial toxicants or therapeutics that can rescue mitochondrial dysfunction. Also, this study demonstrates the usefulness of the SJCRH30 cell line in introducing and modeling a dominant *POLG* mutation. Therefore, we expect SJCRH30 will serve as an excellent cell line background to study additional mitochondrial disease mutations in the future.

## Experimental Procedures

### Construction of the SJCRH30 POLG Y955C cell line using CRISPR-Cas9

SJCRH30 cells (RC13, RMS 13, SJRH30, ATCC CRL2061) were purchased from American Type Culture Collection (ATCC) and grown according to ATCC recommendations. The Stable Cell Line Generation Services from Life Technologies Corporation, operating as the Life Sciences Solutions Group of Thermo Fisher Scientific, was used to optimize SJCRH30 transfections and construct the SJCRH30 *POLG* c.2864A>G/p.Y955C mutant (hereafter *POLG* Y955C) cell line. Briefly, the IVT gRNA was synthesized using the GeneArt Precision gRNA Synthesis Kit. Target F1 (TAA TAC GAC TCA CTA TAG TTC AAC TAC GGC CGC A) and Target R1 (TTC TAG CTC TAA AAC TAG ATG CGG CCG TAG TTG A) primers were used with the 80-base pair CrRNA/Tracr Fragment + the T7 Primer Mix (containing the universal PCR amplification primers) in a PCR assembly reaction to generate the gRNA DNA template. The gRNA DNA template was used in an IVT reaction to make the gRNA, and the gRNA was purified using the MEGAclear Transcription Clean-Up Kit. An asymmetric donor single-stranded DNA (ssDNA) oligonucleotide was synthesized to introduce the *POLG* Y955C mutation *via* the cellular HDR pathway as described (95). The ssDNA sequence used was 5' GOZ GTG TGA GCC GGT GGT TAA ACT GCA TTA GTA AGC GCT CAG CAA AGG GCT GCC CAG CAC CAC AGA TGC GGC CGT AGT TGA AGA TTT TGG CAT GCT CFO G 3′, where F (Phosphorothioate-A), O (Phosphorothioate-C), and Z (Phosphorothioate-T) signify phosphorothioate modifications. This ssDNA produced the highest editing efficiency of two that were tested by on-target NGS, and no off-targets were detected from eight off-target candidates that were screened. SJCRH30 cells were transfected with the IVT gRNA + GeneArt TrueCut Cas9 V2 nuclease + donor ssDNA. On the day of transfection, SJCRH30 cells were harvested, washed once with Dulbecco's phosphate-buffered saline (DPBS) without calcium and magnesium, and suspended in Neon R buffer to 4 × 10^7^ cells/ml (Tube 1). For each transfection reaction, 1 μg of TrueCut Cas9 V2 + 0.3 μg of gRNA + 10 pmoles of donor ssDNA were premixed in a final volume of 5 μl using Neon “R” buffer and then incubated at room temperature for 5 to 10 min to promote the formation of the Cas9-gRNA ribonucleoprotein complex (Tube 2). The tube 2 mixture contained 44% by volume GeneArt TrueCut Cas9 V2 nuclease + IVT gRNA + donor ssDNA and 56% by volume Neon R buffer. The final 10 μl Neon electroporation reaction mixture was made by mixing 5 μl of cells suspended in Neon R buffer (Tube 1) with the contents of Tube 2. This final suspension was gently mixed and electroporated using a Neon Transfection System with a 10-μl tip and electroporation condition number 17. The electroporated cells were immediately added to a well of a 24-well tissue culture plate containing 0.5 ml of prewarmed cell culture medium and incubated at 37 °C, 5% CO_2_. After ∼9 days of growth, clones were isolated by limited dilution cloning (10 × 96-well plates for a stable pool), screened by cell lysis + PCR amplification of a specific flanking *POLG* Y955C region, followed by Sanger sequencing of the PCR products. The primers used for PCR amplification of the *POLG* Y955C mutation were Y955C_F (TGC TTT TCT CAC TCC TGC TTG TC) and Y955C_R (GGT CCT GGG TGT TAA AGT GGA TG). A candidate clone from the Sanger sequencing analysis of 94 samples was subjected to on-target NGS analysis. NGS confirmed the generation of a single clone with 50% HDR resulting in a heterozygous *POLG* Y955C mutation. The clone was expanded and tested for mycoplasma (the results were negative for mycoplasma contamination). Finally, the mutation was reverified by on-target NGS with a 51% conversion frequency of the intended mutation.

### Comparison of SJCRH30 POLG WT and Y955C growth in tissue culture

Proliferating SJCRH30 WT and Y955C mutant cells were grown in RPMI-1640/10% fetal bovine serum (FBS) growth medium, and cell growth and viability were monitored every day over 1 week. Each cell type was initially seeded in fourteen 60-mm tissue culture dishes at 2.5 × 10^4^ cells/cm^2^ and grown at 37 °C, 5% CO_2_. The medium was refreshed every 3 days. Every day, two plates were removed to determine total cell counts (cells/cm^2^) and viability. Each dish was washed with 3 ml of prewarmed DPBS, trypsinized with 0.6 ml 0.25% trypsin for 5 min at 37 °C, and neutralized using 1.2 ml prewarmed RPMI-1640/10% FBS. At early time points, cells were pelleted at 250*g* for 5 min at 4 °C. Then, the supernatant was decanted, and the pellets were suspended in a smaller volume of growth medium to obtain counts of 100 to 300 cells per 1 × 1 mm grid on a hemocytometer. Twenty microliters of the cell suspension was transferred to a microcentrifuge tube, and 20 μl of trypan blue was added. The tube was flicked a few times by fingertip, and 18 μl was loaded into a hemocytometer counting chamber to determine the number of viable (clear) and dead (blue) cells. At least four 1 × 1 mm grids of a hemocytometer counting chamber were counted. The average number of cells per grid was used to calculate the concentration, cells/ml. The cells were pelleted by centrifugation at 250*g* for 5 min, and the supernatant was aspirated and discarded. Next, the cells were gently washed with 0.7 ml DPBS per 1 × 10^6^ viable cells and pelleted by centrifugation, and the supernatant was discarded. Finally, the cells were washed in 0.2 ml of DPBS per 1 × 10^6^ cells and pelleted at 250*g*. The supernatant was removed, and pellets were stored at −80 °C.

### Preparation of whole-cell DNA extracts

WCE DNA samples were prepared from the viable DPBS-washed cell pellets utilizing our in-house DNA extraction method (44), which is based on published work (40). Briefly, a frozen 5 × 10^6^ cell pellet was suspended in 2.0 ml of Proteinase K digestion buffer (PKDB, 100 mM Tris-Cl pH 8.5, 5 mM EDTA, 0.2% SDS, 200 mM NaCl, 0.3 mg/ml proteinase K, 1.1 mM 2-mercaptoethanol) then digested overnight at 55 °C. On the next morning, the digested sample was distributed into four microcentrifuge tubes (0.5 ml in each tube), 10 μl of additional PKDB was added to each tube (0.51 ml total), and the samples were digested for an additional 1 h at 55 °C. Protein and membrane components were precipitated by adding 0.17 ml of 5 M NaCl to each microcentrifuge tube and mixing by inversion for 5 min. Samples were centrifuged at 15,000*g* for 15 min to pellet protein and membrane components, and the supernatant containing nucleic acid was collected and then subjected to ethanol precipitation. After pelleting the nucleic acid samples and washing them with 70% ethanol, the pellets were air dried for 3 h. The nucleic acid samples were resuspended in 1× TE buffer (10 mM Tris-Cl, pH 8.0, 1 mM EDTA) + 1 mM 1,3-dimethylurea (DMU), and the DNA concentration was quantitated by Qubit. Unless otherwise stated, restriction endonuclease digestions and other enzyme treatments were performed according to the manufacturers’ recommendations.

### Flow cytometry

For cell cycle analysis, *POLG* WT and Y955C mutant cells grown in RPMI-1640/10% FBS were transferred to an RPMI-1640/0.5% FBS synchronization medium and seeded at 1 × 10^5^ cells/cm^2^. After 48 h of synchronization at 37 °C, 5% CO_2_, the cells were harvested by centrifugation at 250*g* for 5 min, washed in PBS/6.85 mM EDTA (5 ml per 1 × 10^6^ cells), and then resuspended in PBS/6.85 mM EDTA (0.5 ml per 1 × 10^6^ cells). Next, the cells were fixed in 70% ethanol at 4 °C for at least 2 h (4.5 ml per 1 × 10^6^ cells), harvested by centrifugation at 500*g* for 5 min, and then washed in PBS (5 ml per 1 × 10^6^ cells). Finally, 1 × 10^6^ cells were stained in 0.5 ml PBS/0.02 mg/ml propidium iodide/0.1% Triton X-100/0.2 mg/ml RNase A by incubating at room temperature for 15 min and protected from light. For the preparation of negative control samples, the propidium iodide was not added to the staining solution. The cells were filtered into tubes with filter tops and then analyzed on an Attune NxT Acoustic Focusing Cytometer according to the manufacturer’s recommendations.

### Determination of ddC IC_50_ values

The SJCRH30 cytotoxic effect of ddC was initially estimated *via* percent growth inhibition relative to untreated vehicle control cells. This effect is expressed as the half-maximal inhibitory concentration (IC_50_) or concentration of ddC that reduces the number of viable treated cells by 50% relative to untreated control cells. The day before drug exposure, proliferating SJCRH30 *POLG* WT and Y955C cells in RPMI-1640/10% FBS were separately seeded at 2 × 10^4^ cells/cm^2^ in Falcon 24-well flat-bottom tissue culture plate wells and grown at 37 °C, 5% CO_2_ in a humidified incubator. On the next day, cells in triplicate wells were exposed to RPMI-1640/10% FBS treatment media containing 128, 8, 4, 2, 1, 0.5, 0.25, or 0 μM ddC for 9 days, and the treatment media were refreshed every 3 days. On the day of counting, treatment media were removed. The viable cells remaining in triplicate wells were washed with DPBS, trypsinized, neutralized with their respective treatment medium, pooled together, and then counted using the trypan blue exclusion method as described above. When needed, the cell suspensions were pelleted at 250*g* for 5 min at 4 °C, the supernatant was decanted, and the pellets were suspended in a smaller volume of treatment media to obtain counts of 100 to 300 cells per 1 × 1 mm grid on a hemocytometer. Untreated cell counts (0 μM ddC, vehicle control) were set to 100%, and the IC_50_ values were calculated in Prism 7 using nonlinear regression analysis, the least-squares fit of inhibitor concentration *versus* normalized response.

### ddC treatment of SJCRH30 POLG WT and Y955C cells to determine mtDNA depletion

For each experiment, proliferating SJCRH30 *POLG* WT and Y955C in RPMI-1640/10% FBS growth medium were seeded in fourteen 60-mm tissue culture dishes at 2 × 10^4^ cells/cm^2^ and allowed to adhere to the bottom of the dishes overnight at 37 °C, 5% CO_2_ in a humidified incubator. The ddC treatment medium was prepared by adding ddC to a final concentration of 1 μM in RPMI-1640/10% FBS, and the medium was stored at 4 °C. On the next day, and for each cell type, cells from 2 of the 14 dishes were harvested for timepoint 0 of the experiment. The growth medium was removed from both dishes, and the cells were gently washed with prewarmed DPBS, trypsinized, neutralized with growth medium, and pooled together. The cell suspension was pelleted at 250*g* for 5 min at 4 °C, the supernatant was decanted, and the pellet was suspended in a smaller volume of the growth medium to obtain counts of 100 to 300 cells per 1 × 1 mm grid on a hemocytometer using the trypan blue exclusion method as described above. The cells were centrifuged at 250*g* for 5 min, washed in DPBS (0.7 ml per 1 × 10^6^ cells), and then resuspended in 0.2 ml DPBS per 1 × 10^6^ cells. The tubes were centrifuged at 250*g* for 5 min, the supernatant was aspirated, and the cell pellets were stored at −80 °C.

The RPMI-1640/10% FBS growth medium was replaced with a prewarmed ddC treatment medium for the remaining 12 dishes. The treatment medium was refreshed every 3 days. On days 1 to 6 post treatment, two of each cell culture dish were separately harvested as described above for timepoint 0, except that the ddC treatment medium was used to neutralize the trypsin. The range of cells counted on the hemocytometer was maintained between 100 to 300 cells per 1 × 1 mm grid. The experiments were performed 3 times. Whole-cell DNA was extracted from cell pellets as described above.

### ddC treatment of SJCRH30 POLG WT and Y955C cells to determine mtDNA copy number recovery

For each experiment, proliferating SJCRH30 *POLG* WT and Y955C in RPMI-1640/10% FBS growth medium were seeded in five 100-mm tissue culture dishes at 1 × 10^5^ cells/cm^2^ and allowed to adhere to the bottom of the dishes overnight at 37 °C, 5% CO_2_ in a humidified incubator. The ddC treatment medium was prepared by adding ddC to a final concentration of 1 μM in RPMI-1640/10% FBS, and the medium was stored at 4 °C. On the next day, and for each cell type, cells from one of the five dishes were harvested for timepoint 0 of the experiment. The growth medium was removed from the dish, and the cells were gently washed with prewarmed DPBS, trypsinized, and neutralized with growth medium. The cells were counted using the trypan blue exclusion method. Next, the cells were centrifuged at 250*g* for 5 min, washed in DPBS (4.2 ml per 6 × 10^6^ cells), and then resuspended in 1.2 ml DPBS per 6 × 10^6^ cells. The tubes were centrifuged at 250*g* for 5 min, the supernatant was aspirated, and the cell pellets were stored at −80 °C.

The RPMI-1640/10% FBS growth medium was replaced with prewarmed ddC treatment medium for the remaining four dishes. The cells were exposed to ddC for 24 h. Next, another dish was harvested to obtain cell pellets as described above, except that the treatment medium was used to neutralize the trypsin. For the remaining three dishes, the treatment medium was removed, the cells were gently washed with prewarmed DPBS, and the growth medium was added to allow for recovery of mtDNA replication. On days 1, 3, and 5 of recovery (post treatment), a cell culture dish was harvested as described above for timepoint 0. The experiments were performed twice. Whole-cell DNA was extracted from cell pellets as described above.

### Determination of relative mtDNA copy number

To estimate the mtDNA copy number, WCE DNA was subjected to BamHI digestion. One microgram of each WCE DNA sample was digested with 5 units of BamHI restriction enzyme at 37 °C for 3 h. Next, the WCE DNA digests were loaded onto 1.0% agarose gels in 1× TAE buffer (40 mM Tris, 20 mM acetic acid, 1 mM EDTA) without ethidium bromide and were electrophoresed at 1.1 V/cm for 16 h. The DNA samples were subjected to in-gel fragmentation/depurination, in-gel denaturation, Southern blotting, dual nDNA/mtDNA DIG-labeled probe hybridization (nDNA/RNA18SP4 probe nucleotide positions 101–600; mtDNA-specific probe, GenBank ID MK175431.1 nucleotide positions 168–606), membrane imaging, and band quantification as described (44, 53).

### Characterization of mtDNA topoisomers

The three replicates for each treatment (with or without S1 nuclease) were separate WCE DNA preparations from different passages of cells harvested at day 5 of growth in RPMI-1640/10% FBS growth medium and initially seeded at 2.5 × 10^4^ cells/cm^2^. For 1D-AGE, WCE DNA samples were treated overnight at room temperature with a final concentration of 0.2 mg/ml RNase A. Next, 10 μg of each sample was digested with 50 units of BglII restriction enzyme at 37 °C for 4 h. The BglII-digested WCE DNA was precipitated with a final concentration of 0.1 M sodium acetate in 67% ethanol for 1 h at −20 °C and then centrifuged at 15,000*g* at 4 °C for 15 min. The supernatant was decanted, and the pellet was washed with 1 ml 70% ethanol, then air-dried for 1 h. The dried WCE DNA was dissolved in 50 μl TE + 1 mM DMU and quantitated using a Qubit fluorometer. One microgram of samples was loaded on each lane of 0.4% agarose gel made in 0.5× TBE (44.6 mM Tris, 44.5 mM boric acid, and 1 mM EDTA). For S1 nuclease treatment, 1 μg samples were digested using 2 units of S1 nuclease (Invitrogen) in an 18-μl reaction mixture containing 30 mM sodium acetate (pH 4.6), 145 mM NaCl, 1 mM zinc acetate, and 5% (v/v) glycerol at 37 °C for 30 min. The reaction was stopped by adding 2 μl of 20 mM EDTA and loaded immediately onto the gel. The 1D gel was run for 16 h at 1.85 V/cm, and the buffer chamber was surrounded with ice in a water-tight storage container during electrophoresis. Next, in-gel denaturation of WCE DNA samples, Southern blotting, and hybridizations with the double-stranded DIG-labeled mtDNA-specific probe (nucleotide positions 168–606) were conducted as described (44), with the exception that the gel was left in the gel tray to prevent breakage. For nondenatured 1D gels, the denaturation step was omitted, and the other processing steps were identical to the steps for denatured gels.

2D-AGE was done according to Kolesar *et al.*(40) with slight modifications. WCE DNA samples were prepared, treated with RNase A, digested with BglII, and ethanol precipitated for S1 nuclease digestion as described above for the 1D-AGE method. For each lane of a first dimension 0.4% agarose gel made in 0.5× TBE, 2 μg of a WCE DNA sample was loaded into the lane. For each S1 nuclease–treated WCE DNA sample, 2 μg of DNA was digested using 4 units of S1 nuclease in an 18-μl reaction mixture (30 mM sodium acetate, pH4.6, 145 mM NaCl, 1 mM zinc acetate, 5% glycerol) at 37 °C for 30 min. The reaction was stopped by adding 2 μl of 20 mM EDTA and loaded immediately onto the gel. For the WCE DNA samples that were not treated with S1 nuclease, 2 μg of the samples were digested with BglII and then immediately loaded onto the gel. The 1D gel was run at 1.85 V/cm for 20 h (again, the buffer chamber was surrounded with ice), and the gel was stained in a 0.3 μg/ml ethidium bromide (EtBr) bath for 30 min. For the second dimension, the lane from the first dimension was cut using a razor blade and placed at the top end of a gel casting tray (rotated 90^o^ from the first dimension). The second dimension was caste with 0.4% agarose gel containing 0.3 μg/ml EtBr in 0.5× TBE. The 2D gels were overlayed with 0.5× TBE buffer plus 0.3 μg/ml EtBr and electrophoresed at 1.85 V/cm for 20 h, and the buffer chamber was surrounded with ice in a water-tight storage container during electrophoresis. Gels were subjected to denaturing conditions, Southern blotting, and nonradioactive probe hybridization as described above for 1D gels.

### Two-dimensional neutral agarose gel electrophoresis

Five micrograms of WT and Y955C WCE DNA in TE + DMU were separately digested with HincII according to manufacturer’s recommendations. Next, the restriction endonuclease–digested samples were subjected to 2DNAGE and Southern blotting and probed with a mtDNA-specific probe (positions 37–611) as described (96). The mtDNA probe is specific to an ∼3.9-kb subgenomic mtDNA HincII restriction fragment harboring the heavy (H) strand origin of replication (O_H_). The blots were exposed to film for 8 days, and images were collected.

### Probe hybridization conditions for mtDNA restriction enzyme mapping

The restriction endonuclease mapping experiments used nonradioactive single-stranded covalently linked DIG probes specific for mtDNA L- or H-strands. For each restriction endonuclease digest, 3 μg of WCE DNA was digested with 15 units of restriction enzyme for 16 h, and then digests were run on a 1% agarose gel followed by Southern blotting and probe hybridization. The probes were synthesized by IDT (Integrated DNA Technologies) and contained 5' DIG NHS and 3′ DIG NHS esters. The single-stranded probes were specific to *ND1* and *ND4* genes: (1) *ND1* heavy, /5′DIGN/GAA TGG GTA CAA TGA GGA GTA GGA GGT TGG/3′DIG_N/; (2) *ND1* light, 5′DIGN/CCA ACC TCC TAC TCC TCA TTG TAC CCA TTC/3′DIG_N/; (3) *ND4* heavy, /5′DIGN/GAG GAT TAT GAT GCG ACT GTG AGT GCG TTC/3DIG_N/; and (4) *ND4* light, /5′DIGN/GAA CGC ACT CAC AGT CGC ATC ATA ATC CTC/3′DIG_N/.

For the prehybridization step, blots were placed in a 35 × 150 mm hybridization bottle containing 6 ml of prehybridization solution and incubated for 2 to 3 h at 46 °C in a hybridization oven rotary wheel carousel at maximum rpm. The prehybridization solution was decanted, and 6 ml of prewarmed probe (200 ng/ml) was added to the hybridization bottle and incubated overnight at 46 °C in the hybridization oven at maximum rpm. On the next day, the probe solution was decanted, and blots were washed twice with 2× SSC + 0.1% SDS for 5 min at room temperature. Next, the blots were washed with 0.5× SSC + 0.1% SDS twice at 50 °C for 15 min. The remaining steps were done as described (44).

### Long-range PCR for analysis of mtDNA deletions

The forward and reverse primers were mt10F, 5′TCT ATC ACC CTA TTA ACC ACT CAC GGG AGC T and mt16496R, 5′CGG ATA CAG TTC ACT TTA GCT ACC CCC AAG TG and were used to generate an amplicon that was nearly the size of the entire mtDNA genome (∼16.5 kb). The long-range PCR was performed using Phusion High Fidelity PCR master mix (Thermo Fisher Scientific) and 110 ng of WCE DNA isolated from SJCRH30 *POLG* WT or Y955C cells as the template in a 50-μl PCR reaction. An initial 30-s incubation at 98 °C was followed by 32 cycles of PCR with 20 s of denaturation at 98 °C and 9 min of annealing and extension at 72 °C. The reaction was completed by one cycle of final extension at 72 °C for 12 min; 46 μl of each PCR reaction was analyzed on 1% agarose gels with both exACTGene 1 kb Plus DNA ladder (Fisher BioReagents) and Lambda DNA/HindIII Marker (Thermo Fisher Scientific) used as molecular weight standards. The gel was run for 1 h 40 min at 5.3 V/cm, stained in a bath of 0.5 μg/ml EtBr, and imaged on a Syngene G:Box Chemi XX9 with a 9.0-megapixel, 16-bit, ultra-cooled CCD camera.

### Seahorse XFp bioenergetic analysis

The Seahorse extracellular flux analyzer was used to measure bioenergetics according to the manufacturer’s recommendations. Briefly, the SJCRH30 *POLG* WT and Y955C mutant cells were initially seeded in RPMI-1640/10% FBS at 2.5 × 10^4^ cells/cm^2^, and the cells were separately harvested on the third day of growth. Various cell densities were seeded into Seahorse cell culture miniplate wells, and the cells were allowed to adhere to the well bottoms. The miniplates were incubated overnight at 37 °C, 5% CO_2_. Three WT seeding densities were tested in 180 μl Mito Stress test assay medium: 1.9 × 10^5^, 1.3 × 10^5^, and 8.9 × 10^4^ cells/cm^2^ (2 × 10^4^, 1.34 × 10^4^, and 9.4 × 10^3^ cells per well, respectively). The Mito Stress test assay medium consisted of XF Base Medium (0 mM glucose, Agilent Technologies) supplemented with 1 mM pyruvate, 2 mM glutamine, and 10 mM glucose. The assay medium pH was adjusted to 7.4 ± 0.1 using 0.1 N NaOH, then the medium was sterilized by filtering through a 0.2-μm filter. On the next day, the basal oxygen consumption rate (OCR) and extracellular acidification rate (ECAR) values were determined by running the cell culture miniplates on the Seahorse XFp. Basal OCR values ranged from 69 to 148 pmol O_2_/minute, and basal ECAR values ranged from 24 to 35 mpH/minute. Based on these seeding density tests, the cell culture miniplates were seeded using 1.9 × 10^5^ viable proliferating WT or mutant cells/cm^2^ in subsequent experiments.

Oligomycin and FCCP dose responses were tested consistent with our previous report to ensure optimal inhibition of ATP synthase (complex V) and dissipation of the proton motive force, respectively (32). Briefly, stock solutions of stressors were made and diluted in the Mito Stress test assay medium. To determine the optimal concentration of oligomycin, three different concentrations were loaded into the XFp sensor cartridge, and Mito Stress tests were performed. Twenty microliters of 5 μM (wells B & C), 10 μM (wells D & E), and 20 μM oligomycin (wells F & G) were separately added into each sensor cartridge port A to obtain final well concentrations of 0.5, 1, and 2 μM when diluted into 180 μl Mito Stress test assay medium. For *POLG* WT SJCRH30, maximal decreases in OCRs that did not alter cell morphology or adherence to miniplate wells were observed with 1 μM oligomycin; hence, this concentration was used in subsequent tests. Similarly, five different concentrations of FCCP were tested, and a final concentration of 1 μM FCCP gave the strongest dose–response (increase in OCR values) and was used for subsequent experiments. Finally, for Mito Stress tests, final concentrations of 0.5 μM rotenone and 0.5 μM antimycin A were used to optimally inhibit OXPHOS complexes I and III. Therefore, for Mito Stress tests, 1.9 × 10^5^ viable proliferating WT or mutant cells/cm^2^ in miniplate wells were treated sequentially with 1 μM oligomycin, 1 μM FCCP, and 0.5 μM antimycin A + 0.5 μM rotenone. For side-by-side comparisons of WT and Y955C cell mitochondrial function, each cell type was seeded in triplicate into miniplates wells. After completion of the Mito Stress test, cells were washed with prewarmed DPBS, incubated overnight at -80 °C, then lysed with RIPA lysis buffer supplemented with HALT protease inhibitor cocktail. The total cellular protein content in each miniplate well was measured using the bicinchoninic acid (BCA) protein assay kit, and OCR and ECAR values were normalized to total cellular protein for the determination of bioenergetic parameters as described (32).

For the ECAR Stress tests, target compound stock solutions and the glucose stock solution were made as described (32). The ECAR Stress test assay medium consisted of XF Base Medium (Agilent Technologies) supplemented with 2 mM glutamine. The ECAR Stress test assay medium pH was adjusted to 7.4 ± 0.1, and the medium was sterilized as described above for the Mito Stress test assay medium. Briefly, 20 μl of 100 mM glucose was loaded into port A, a 160 μM oligomycin stock solution was diluted 1 in 16 (to 10 μM), and 22 μl was loaded into port B, and 25 μl of 500 mM 2-DG was loaded into port C (32). For side-by-side comparisons, each cell type was seeded in triplicate into miniplates wells in 180 μl ECAR Stress test assay medium, and ECAR Stress tests were performed. Therefore, 1.9 × 10^5^ viable proliferating WT or mutant cells/cm^2^ in miniplate wells were treated sequentially with 10 mM glucose, 1 μM oligomycin, and ∼51 mM 2-DG. Following the ECAR Stress tests, cells were washed and lysed, and OCR and ECAR values were normalized to total cellular protein as described above for Mito Stress tests. Bioenergetic parameters were calculated as described (32).

SJCRH30 WT and Y955C cells were forced to rely on mitochondrial OXPHOS by substituting glucose for galactose in an RPMI-1640/10% dialyzed FBS growth medium lacking sodium pyruvate. Both cell types were seeded at 2.5 × 10^4^ cells/cm^2^ and passaged every 3 to 4 days. The cells were grown and passaged in the RPMI-1640/10% dialyzed FBS medium for 19 days. Mito Stress tests were performed on days 12, 15, and 19 using passages 8, 9, and 11 for WT and 7, 8, and 10 for Y955C. Mito Stress tests were performed as described above except that 9.4 × 10^4^ cells/cm^2^ were seeded in the Seahorse cell culture miniplates, and 2 μM FCCP was used.

### Live-cell fluorescence microscopy imaging of mitochondrial nucleoids

For fluorescent imaging, 200,000 cells were seeded in 35-mm MatTek cell culture dishes containing 2.5 ml of RPMI-1640/10% FBS growth medium. Plates were incubated overnight in a 37 °C, 5% CO_2_ incubator. On the next day, the growth medium was gently removed and replaced with 2.5 ml of growth medium plus 25 nM of MitoTracker Red CMXRos to stain mitochondria with red fluorescence, and the dishes were incubated for 3 min at 37 °C, 5% CO_2_. After the incubation, the cells were gently washed twice with 2 ml of prewarmed Hank’s balanced salt solution (HBSS), overlayed with 2.5 ml of HBSS, and red fluorescent images were immediately collected. Images were collected using a Leica DMi8 epifluorescence microscope with a Lumencor SOLA light engine, a 63×, 1.4 numerical aperture objective, a TXR (red fluorescence) filter cube (Excitation: 560/40; Dichroic: 585; Emission: 630/75; 250 ms exposure time), and a Hamamatsu ORCA-Flash4.0 V2 digital scientific complementary metal-oxide-semiconductor (sCMOS) camera. For PicoGreen staining of cellular DNA, growth medium was removed from MatTek dishes, and 2.5 ml of medium plus 3 μl/ml of Quant-iT PicoGreen dsDNA Reagent was gently overlayed. Next, the dishes were incubated for 1 h at 37 °C in a 5% CO_2_ incubator. After the incubation, the medium was removed, and the cells were gently washed twice with 2 ml of HBSS. The dishes were overlayed with 2.5 ml of HBSS, and images were immediately collected as described above but this time using a GFP (green fluorescence) filter cube (Excitation: 470/40; Dichroic: 495; Emission: 525/50; 400 ms exposure time). For dual stain imaging with MitoTracker Red and PicoGreen, the cells were initially stained with 25 nM MitoTracker Red in the growth medium for 3 min, the dishes were washed twice with HBSS, and then PicoGreen staining was carried out as described above. The dual-stained images were instantly collected in sequence using differential interference contrast (channel 1) followed by red fluorescence (channel 2) and green fluorescence (channel 3). For both the *POLG* WT and Y955C mutant, the experiment was repeated twice using different passages of cells on different days. For each experiment, unstained mock-treated dishes were run in parallel and served as negative controls for day-to-day green and red autofluorescence, which were negligible. Two images collected on different days for each cell type were analyzed using Leica LAS X software. Linear adjustments were made in LAS X, and the same software was used to generate scale bars. For each image, large PicoGreen-stained nuclei in channel 3 (that are excluded from the region of MitoTracker Red staining seen in channel 2) were counted in 32 cells. In parallel, green-stained cytoplasmic mtDNA nucleoid puncta in channel 3, which localized with red-stained mitochondria in channel 2, were counted in the same 32 cells, and data are reported as PicoGreen puncta per nuclei per focal plane.

### MitoTracker Red CMXRos–stained mitochondria measurements

Mitochondrial imaging analysis was done using the Mitochondrial Analyzer plugin for ImageJ/Fiji on a Dell Precision M4800 laptop (52). For each cell line, the experiment was repeated twice on different days. We analyzed two images from each experiment (n = 4 replicate images for each cell line) containing at least 111 cells. MitoTracker Red–stained images were deconvoluted using Leica LASX software and then exported as TIFFs. The TIFFs were separately opened in Fiji, converted to 8 bit, and preprocessed. Preprocessing was done using Mitochondrial Analyzer to subtract background (radius = 1 μm), sigma filter plus (to reduce noise but preserve edges), enhance local contrast (max slope = 2), and gamma correction (value = 0.8). Multiple thresholding conditions were tested using the 2D Threshold Optimize option, and the adaptive threshold method with weighted mean was used with a block size of 1.25 μm and C values of 1 or 2 to identify mitochondria. Also, the despeckle and remove outliers postprocessing commands were selected. Following image thresholding, 2D analysis was performed on a per-cell (image) basis. The data for each image were normalized to values per cell. For example, the “count” value for mitochondria in an image was divided by the number of cells per image.

### Isolation of mitochondria

Mitochondria were isolated from WT passage 15 and Y955C passage 14 cells based on the protocol developed by Clayton and Shadel (97). Briefly, at least four 150-mm tissue culture dishes were seeded with 2.5 × 10^4^ cells/cm^2^ in RPMI-1640/10% FBS, and the cells were allowed to grow for 5 days at 37 °C, 5% CO_2_. The medium was refreshed on the third day of growth. On the fifth day, the cells were harvested by washing each dish with 20 ml of prewarmed DPBS, trypsinizing with 3 ml 0.25% trypsin for 5 min at 37 °C, and neutralizing the trypsin with 6 ml prewarmed RPMI-1640/10% FBS. The cells were sequentially washed with 50 ml DPBS per 1 × 10^8^ cells followed by 5 ml DPBS and then immediately used to isolate mitochondria.

The following steps were performed on ice. For a 0.5-ml wet weight pellet, the cells were suspended in 5 ml ice-cold calcium reticulocyte standard buffer (10 mM NaCl, 1.5 mM CaCl_2_, 10 mM Tris-Cl, pH7.5) and were transferred to a Dounce homogenizer. The cells were allowed to swell for 10 min and then broken open with 33 strokes of a tight-fitting pestle. Next, 3.7 ml of 2.5× mannitol sucrose homogenization buffer (2.5× MS; 525 mM mannitol, 175 mM sucrose, 2.5 mM EDTA, 12.5 mM Tris-Cl, pH 7.5) was quickly added to the lysate. The pestle was removed, and the top of the Dounce homogenizer was sealed with Parafilm and mixed by inverting it three times. The homogenate was transferred to a centrifuge tube, and the volume was adjusted to 13.8 ml with 1× MS buffer (210 mM mannitol, 70 mM sucrose, 1 mM EDTA, 5 mM Tris-Cl, pH 7.5). Nuclei were pelleted by three successive sedimentations at 1300*g* for 5 min. Each time the nuclei were pelleted, the supernatant containing the mitochondria was gently aspirated using a serological pipette without disturbing the pellet. The final mitochondria supernatant was transferred to a new centrifuge tube and pelleted at 16,000*g* for 15 min. The supernatant was discarded, and the mitochondrial pellet was resuspended in an equivalent volume of 1× MS buffer. The mitochondrial protein content in each sample was measured using the BCA protein assay kit, and aliquots of the mitochondria were stored at −80 °C. For 1.3 × 10^8^ WT and *POLG* Y955C cells, 3.5 and 2.1 mg of mitochondria were obtained (27 pg of mitochondrial protein per WT cell and 16 pg of mitochondrial protein per Y955C cell).

### Preparation of protein extracts

To prepare a mitochondrial sample for sodium dodecyl sulfate polyacrylamide gel electrophoresis (SDS-PAGE), a 40-μl aliquot was taken from -80 °C and thawed on ice. The mitochondria were lysed in ice-cold RIPA lysis buffer (50 mM Tris-Cl pH 8.0, 150 mM NaCl, 1% Igepal CA-630, 0.5% deoxycholate, 0.1% sodium dodecyl sulfate) supplemented with a 1 in 101 dilution of HALT protease inhibitor cocktail (Thermo Fisher Scientific). The WT and Y955C mitochondria (12.6 and 12.9 μg/μl protein, respectively) were separately diluted down to 5 μg/μl protein in RIPA lysis buffer + HALT. Next, the samples were pipetted up and down to mix and then underwent four successive vortex cycles for 30 and 10 s on ice. The samples were mixed end-over-end for 15 min at 4 °C, and then the four successive cycles of vortex and incubation on ice were repeated. Next, the samples were pipetted up and down 10 times, Laemmli’s sample buffer was added to bring the final protein concentration to 3.33 μg/μl, and then the samples were boiled at 95 °C for 5 min. The samples were stored at −80 °C, thawed, and heated at 65 °C for 5 min before loading the gel.

To prepare a whole-cell extracted protein sample for SDS-PAGE, a prefrozen 5 × 10^6^ DPBS washed cell pellet was thawed on ice and suspended in 150 μl cold RIPA lysis buffer + HALT. Next, the samples were pipetted up and down to mix and then underwent four successive vortex cycles for 30 and 10 s on ice. The samples were mixed end-over-end for 15 min at 4 °C, and then the four successive cycles of vortex and incubation on ice were repeated. Next, the samples were pipetted up and down 10 times and vortexed for 10 s. Following protein concentration determination using the BCA assay, the whole-cell extracted protein concentration was adjusted to 4.5 μg/μl using RIPA + HALT. Laemmli’s sample buffer was added to bring the final protein concentration to 3.0 μg/μl, and the samples were boiled at 95 °C for 5 min. The samples were stored at −80 °C, thawed, and heated at 65 °C for 5 min before loading the gel.

### SDS-PAGE and Western blotting

Fifteen percent SDS-PAGE resolving/5% stacking gels were made, according to Green and Sambrook (98). Fifty micrograms of cell or mitochondrial extract was loaded per lane. The bands detected by Western blotting (immunoblotting) were normalized to total cellular protein by adding 0.5% 2,2,2-trichloroethanol to the resolving gel as described (99). Using the Bio-Rad Mini-PROTEAN Tetra Cell system, the gels were run in 1× electrode running buffer (25 mM Tris, 192 mM glycine, 0.1% SDS, pH 8.3) at 100 V for 20 min. Subsequently, the voltage was increased to 200 V for 1 h. The gels were removed from the glass plates and exposed to UV using the Syngene G:Box Chemi XX9 transilluminator for 5 min to quantify 2,2,2-trichloroethanol-stained total cellular protein on the nitrocellulose membrane (see below). Next, the gels were equilibrated in transfer buffer (25 mM Tris, 195 mM glycine, 10% 200 proof ethanol) for 15 min. The proteins were transferred to nitrocellulose membranes at 110 V for 2 h in a precooled transfer buffer at 4 °C. After electroblotting, the nitrocellulose membranes were imaged using the Syngene G:Box Chemi XX9 transilluminator for 20 s, and a digital image was captured for normalization.

The nitrocellulose membranes were washed for 5 min in TBST (136.9 mM NaCl, 2.68 mM KCl, 24.77 mM Tris, pH 7.4, 0.1% Tween 20) and blocked for 1 h in TBST + 5% nonfat instant dry milk at room temperature. The blots were washed for 2 min in TBST and then incubated for 16 h in either a 1/500 dilution of the mouse monoclonal total OXPHOS human Western blot antibody cocktail or a 1/1000 dilution anti-mt-ND3 rabbit polyclonal antibody (ABCAM). Both antibodies were diluted in TBST + 5% bovine serum albumin. Next, the blots were washed 3 times with TBST buffer for 10 min, followed by incubation in their respective 1/3000 dilutions of either goat anti-rabbit IgG (H + L) alkaline phosphatase–conjugated antibody (Bio-Rad) or 1/3000 dilution of goat anti-mouse IgG (H + L) alkaline phosphatase–conjugate (Bio-Rad) in TBST + 5% nonfat instant dry milk. After three 10-min washes in TBST, the bands were visualized by chemiluminescence with CDP-Star (Roche) using the Syngene G:Box Chemi XX9 gel documentation system.

### Molecular modeling of p140 Y955C

The p140 Y955C catalytic subunit PDB structural model was generated using the 4ZTZ PDB file at the Missense3D database. “Position on 3D Structure” was selected and the 4ZTZ PDB code, the PDB chain ID (A), residue ID (955), WT amino acid tyrosine, and mutant amino acid residue cysteine were entered into the variant information form at http://missense3d.bc.ic.ac.uk/∼missense3d/ (100). To show the primer-template next to the p140 Y955C disease variant structure in Figure 1*A* (bottom right), the Missense3D generated “mutant PDB structure” was opened in PyMOL along with the original 4ZTZ PDB file. First, hide everything was selected for 4ZTZ, and second, the two DNA strands were selected, and show as sticks was chosen. This process was repeated to show the Mg^2+^ ions as spheres and dCTP as sticks.

### Identification of quadruplex forming potential mtDNA sequences

The quadruplex-forming potential sequences in the mtDNA NC_012920 H and L strands were estimated using the online QGRS (Quadruplex forming G-Rich Sequences) Mapper, a web-based server for predicting G-quadruplexes (G4s), https://bioinformatics.ramapo.edu/QGRS/analyze.php (101). The search parameters were QGRS Max length 33, Min G-group size 2 (*e.g.*, 2GQH, Figure 6*B*) or 3 (*e.g.*, 3GQH, Figure 6*B*), and loop size 1 to 36.

### Statistical analyses used

All data presented are mean values ± standard deviations (SDs). Statistical significance between two parametric groups was determined using a Student’s or a Welch’s *t* test, and significance between two nonparametric groups was conducted using a Mann–Whitney U test. Comparisons of more than two groups of parametric data were assessed by one-way analysis of variance (ANOVA) followed by Tukey’s test or Welch’s ANOVA with Dunnett T3 post hoc test. Comparisons of greater than two groups of nonparametric data were assessed by Kruskal–Wallis tests followed by Dunn’s post hoc test. For the analysis of mtDNA copy number in Figure 7, statistical significance was determined by a two-way ANOVA with a Tukey’s multiple comparison test. *p*-Values less than 0.05 were considered significant.

## Data availability

All the data described herein are within this article. Limited amounts of the cell line may be made available upon request with restrictions. Other reagents, plasmids, etc., are available upon request by contacting the corresponding author M. J. Y.

## Supporting information

This article contains supporting information.

## Conflict of interest

The authors declare that they have no conflicts of interest with the contents of this article.

## Supporting information

Supplementary Figure 1 SJCRH30 *POLG* WT and Y955C cells have similar cell-cycle phases. Using flow cytometry, cells were stained with propidium iodide (PI) to segregate cells into the G_0_/G_1_, S, and G_2_/M phases. Cells in G_0_/G_1_ and G_2_M are seen as separate peaks on the left (red) and right (blue). The population of cells between the G_0_/G_1_ and G_2_/M peaks represents cells in the S phase (green). Cells in the G_2_/M phase have double the ploidy/DNA content/PI fluorescence intensity of cells in the G_0_/G_1_ phase. FSC-H, forward scatter height; SSC-H, side scatter height; SSC-A, side scatter area; R1, gate 1; R2, gate 2; BL2, blue laser (488 nm). The experiments were repeated twice on different days using different passages of cells, and percent values for the different phases are reported as mean ± SDs from n ≥ 4 (≥ duplicate for each experiment). Representative graphs from a WT and Y955C experiment are shown. The analyses were done using an Attune NxT Acoustic Focusing Cytometer

Figure S2 Restriction endonuclease mapping of SJCRH30 *POLG* WT and Y955C mtDNA. For each restriction endonuclease digest, 3 μg of WCE DNA was digested with 15 units of restriction enzyme for 16 h, and then digests were run on a 1% agarose gel followed by Southern blotting and probe hybridization. mtDNA restriction fragments were detected with either an *ND1* light (L)-strand (left) or *ND4* L-strand (right) single-stranded (ss) DIG-labeled probe

Figure S3 The SJCRH30 *POLG* Y955C cell line harbors additional mtDNA topoisomers that are sensitive to S1 nuclease. Samples were treated with RNase A, digested with BglII to fragment nDNA (but not mtDNA), then ethanol precipitated and suspended in 1× TE + 1 mM DMU. The blots shown are the additional replicate blots that match those shown in Figure 8. See Figure 8 for details

Figure S4 Additional two-dimensional agarose gel electrophoresis (2D-AGE) analysis of SJCRH30 *POLG* WT and Y955C mtDNA topoisomers. See Figure 9 for details
