# Supplementary figures and images for: Heterozygous p.Y955C mutation in DNA polymerase γ leads to alterations in bioenergetics, complex I subunit expression, and mtDNA replication
Source: J Biol Chem. 2022 Jun 24;298(8):102196. doi: 10.1016/j.jbc.2022.102196 (PMC9307957; doi:10.1016/j.jbc.2022.102196)

All Events WT

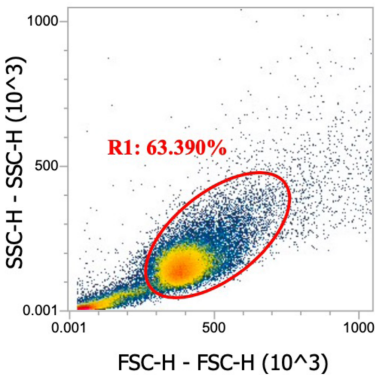

R1 WT

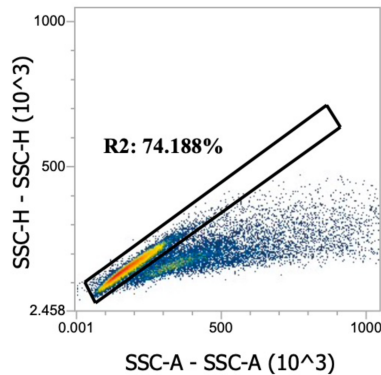

R2 WT

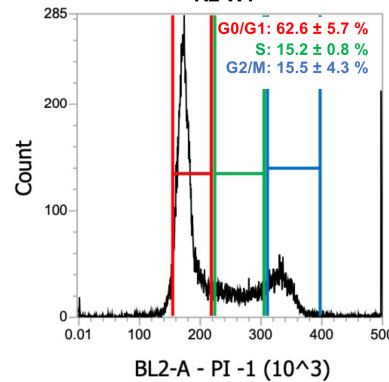

All Events Y955C

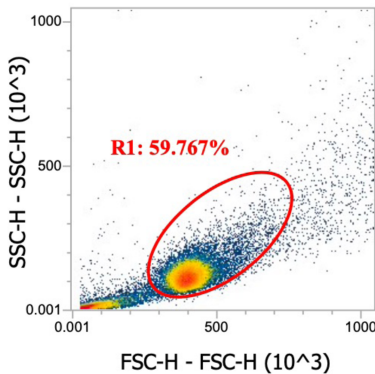

R1 Y955C

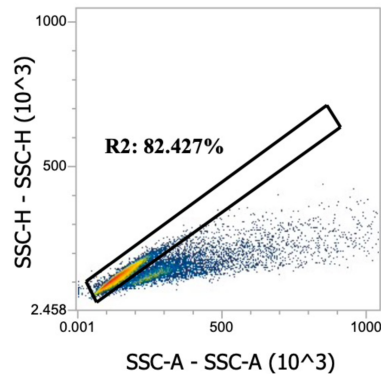

R2 Y955C

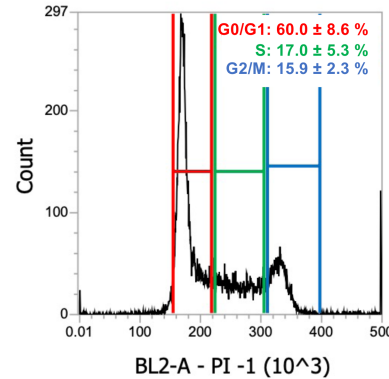

Supplement: Supplementary Figure 1 — SJCRH30 POLG WT and Y955C cells have similar cell-cycle phases. Using flow cytometry, cells were stained with propidium iodide (PI) to segregate cells into the G0/G1, S, and G2/M phases. Cells in G0/G1 and G2M are seen as separate peaks on the left (red) and right (blue). The population of cells between the G0/G1 and G2/M peaks represents cells in the S phase (green). Cells in the G2/M phase have double the ploidy/DNA content/PI fluorescence intensity of cells in the G0/G1 phase. FSC-H, forward scatter height; SSC-H, side scatter height; SSC-A, side scatter area; R1, gate 1; R2, gate 2; BL2, blue laser (488 nm). The experiments were repeated twice on different days using different passages of cells, and percent values for the different phases are reported as mean ± SDs from n ≥ 4 (≥ duplicate for each experiment). Representative graphs from a WT and Y955C experiment are shown. The analyses were done using an Attune NxT Acoustic Focusing Cytometer [file mmc1.pdf]

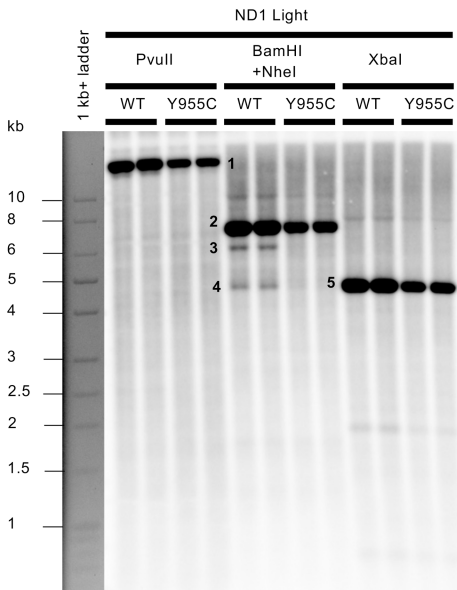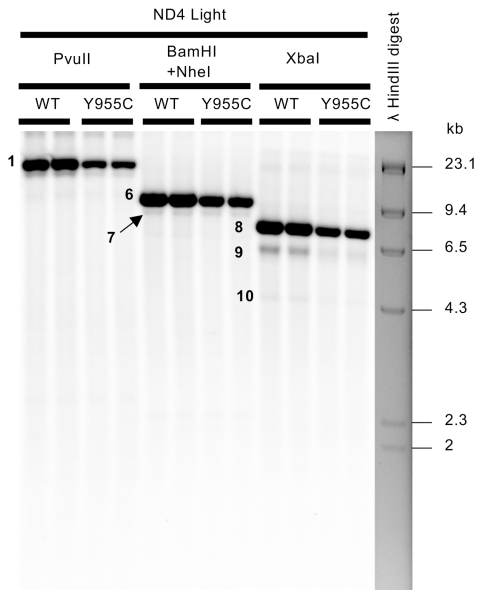

Supplement: Figure S2 — Restriction endonuclease mapping of SJCRH30 POLG WT and Y955C mtDNA. For each restriction endonuclease digest, 3 μg of WCE DNA was digested with 15 units of restriction enzyme for 16 h, and then digests were run on a 1% agarose gel followed by Southern blotting and probe hybridization. mtDNA restriction fragments were detected with either an ND1 light (L)-strand (left) or ND4 L-strand (right) single-stranded (ss) DIG-labeled probe [file mmc2.pdf]

WT untreated

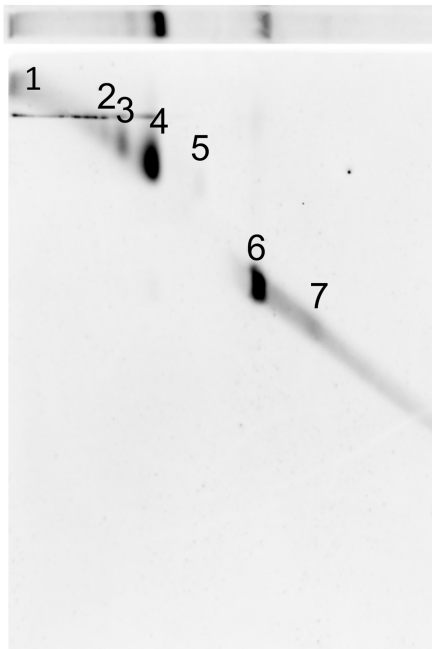

Y955C untreated

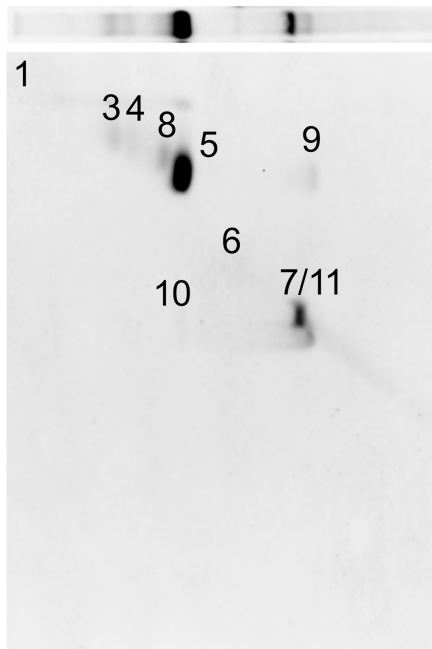

WT S1 nuclease treated

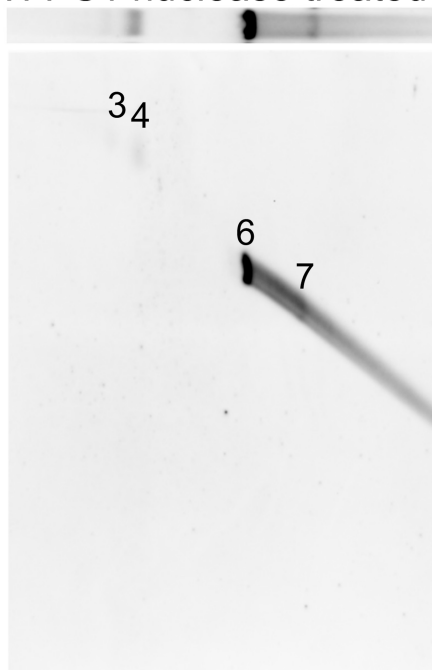

Y955C S1 nuclease treated

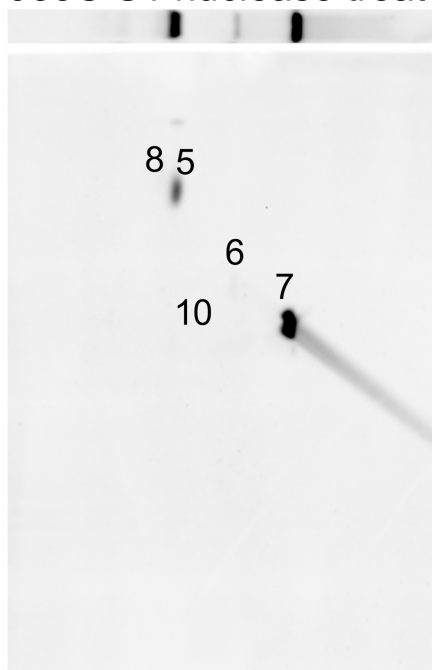

Supplement: Figure S4 — Additional two-dimensional agarose gel electrophoresis (2D-AGE) analysis of SJCRH30 POLG WT and Y955C mtDNA topoisomers. See Figure 9 for details [file mmc4.pdf]
