# Supplementary material for: Heterozygous p.Y955C mutation in DNA polymerase γ leads to alterations in bioenergetics, complex I subunit expression, and mtDNA replication
Source: J Biol Chem. 2022 Jun 24;298(8):102196. doi: 10.1016/j.jbc.2022.102196 (PMC9307957; doi:10.1016/j.jbc.2022.102196)
Supplement: Figure S3 — The SJCRH30 POLG Y955C cell line harbors additional mtDNA topoisomers that are sensitive to S1 nuclease. Samples were treated with RNase A, digested with BglII to fragment nDNA (but not mtDNA), then ethanol precipitated and suspended in 1× TE + 1 mM DMU. The blots shown are the additional replicate blots that match those shown in Figure 8. See Figure 8 for details [file mmc3.pdf]

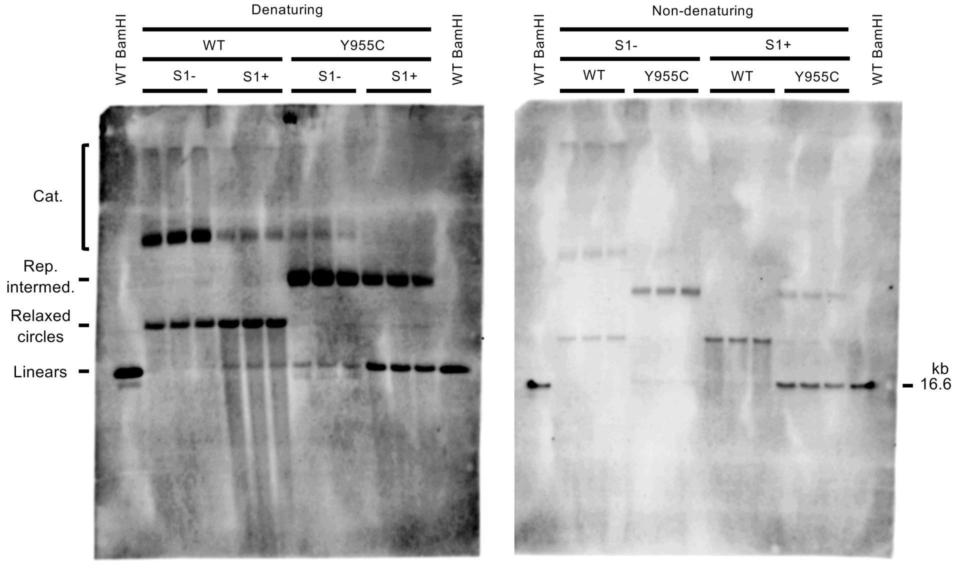
Denaturing
WT
Y955C
S1-
S1+
S1-
S1+
WT BamHI
WT BamHI
Non-denaturing
S1-
S1+
WT
Y955C
WT
Y955C
WT BamHI
WT BamHI
Cat.
Rep. intermed.
Relaxed circles
Linears
kb
16.6
